# Automated Digital Discovery and Synthesis of CuO-Based Nanoparticle Heterostructures for Catalysis

**DOI:** 10.1021/acsami.5c13709

**Published:** 2025-10-11

**Authors:** Daniel Hervitz, Yibin Jiang, Daniel Salley, Mark McNulty, Philip. J Kitson, Leroy Cronin

**Affiliations:** School of Chemistry, 3526 The University of Glasgow, University Avenue, Glasgow G12 8qq, U.K.

**Keywords:** copper oxide, nanoparticles, nanoparticle heterostructure, automation, digital chemistry, self-assembly, photodegradation

## Abstract

The discovery and synthesis of composite nanomaterials often rely on molecular self-assembly and crystallization, posing significant challenges due to the vast chemical space and the irreproducibility of experimental methods. We present a programmable robotic platform, controlled by the universal Chemical Description Language (χDL), that enables the solid-phase synthesis of composite nanomaterials. In addition to synthesis, the platform validates its catalytic performance through an automated workflow. This platform enables open-ended exploration of composition-morphology-activity relationships, with high accuracy and reproducibility, while also reducing synthesis time and cost. In this study, we are moving beyond the colloidal, plasmonic-focused systems previously explored in robotic platforms to the discovery, synthesis, and catalytic properties of CuO-based nanomaterials, such as CuO-Au and CuO-Ag_2_O NP heterostructures that show good reproducibility across repeated syntheses. Remarkably, even at very low metal loadings, as confirmed by ICP (Au wt % = 0.06%, Ag wt % = 0.03%), the heterostructures exhibited enhanced photodegradation efficiency of the dye Methyl Green (MG) compared with pristine CuO. The degradation yield increased from 45 ± 2% for pristine CuO to 57 ± 3% for CuO-Au and 65 ± 2% for CuO-Ag_2_O, as observed through real-time UV–vis spectroscopy. Additionally, a kinetic assay of the synthesis process provided insights into the self-assembly mechanism, highlighting the interactions between the core material (CuO NPs) and the surface coatings (Au or Ag_2_O). This work demonstrates a shift from traditional manual experimentation to programmable, data-driven workflows, highlighting both the progress and the remaining challenges in the automation of solid-phase nanomaterial synthesis in the field of materials science.

## Graphical abstract

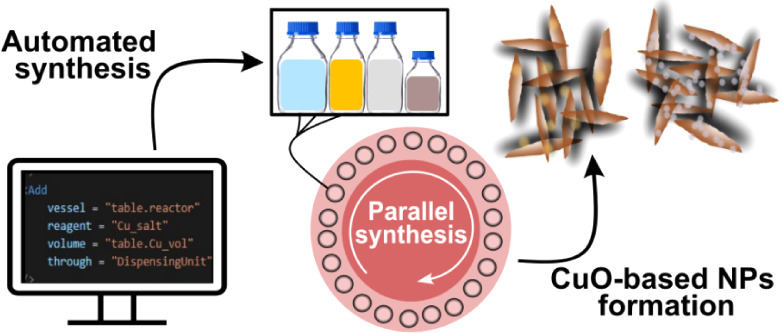

## Introduction

In recent years, there has been intense interest surrounding the controlled synthesis and fabrication of advanced nanostructures with unique properties resulting from their structural characteristics.^3–6^ Nanoparticle heterostructures are defined as materials that combine a nanoparticle’s core material with a surface coating or decoration of a different material, such as a polymer, single atom, or atomic cluster. This can enable synergistic effects that improve material properties, such as enhanced surface reactivity, improved charge transfer, and tunable particle size^7–10^ These particles are classified as innovative advanced nanomaterials, with applications found in various fields, including optics, energy conversion, sensors, and catalysis.^11–13^

As core materials, metal oxide semiconductors possess unique structural characteristics that make them highly valuable for catalytic and photocatalytic applications. Their performance is strongly influenced by factors such as size, morphology, and crystal structure, enabling their effective use in processes including CO_2_ reduction, pollutant degradation, and water splitting.^14–18^ Among the metal oxide semiconductors, CuO- a p-type oxide with high photosensitivity, physicochemical stability, and a band gap of 1.2–1.7 eV, has exhibited potential in visible-light-driven photocatalytic reactions.^19,20^ However, CuO’s photocatalytic performance is limited by the quick recombination of the electron–hole pair, which decelerates photocatalytic activity.^21–23^ To overcome this limitation, plasmonic photocatalysts comprising noble metals (typically Au, Ag, Pd, or Pt) have attracted attention due to the surface plasmon resonance (SPR) effect. This can promote charge separation and energy transfer to enhance the light absorption of the material, leading to suppression of photoexcited charge carriers.^24–28^ Therefore, hybridizing plasmonic metals (*e.g*., Au, Ag) with a CuO semiconductor to form metal–semiconductor nanoparticle heterostructures has been proposed as a promising way to enhance photocatalytic efficiency.

There are several challenges in producing these materials, including procedures that require high temperatures, the use of multiple reagents, and tedious synthesic procedures.^28–30^ It is therefore of great interest to develop a reproducible and effective platform capable of controlling diverse synthetic conditions. Recently, there has been a rapid growth in the development of robotic synthesis platforms for the discovery and exploration of new compounds, from small molecules and peptides to nanomaterials, providing an autonomous workflow that emphasizes optimization through closed-loop design or machine learning.^1,2,31^ However, automated synthesis of solid-phase materials remains underexplored. Therefore, we developed a programmable modular system with hardware capable of performing the fundamental processes of chemical synthesis by using the Chemical Description Language χDL. At the core of χDL are the unit operation steps that represent a chemical synthesis as a sequence of discrete operations that enable automated synthesis and characterization. The use of a machine-readable, standardized language enables researchers to adjust key reaction parameters, such as precursor concentrations, reducing agents, and stabilizers across nanomaterial discovery spaces, while simultaneously allowing in-line evaluation of catalytic activity. This approach enhances efficiency by reducing laboratory time and synthesis costs while ensuring reliable data.

In this study, we present the development of a fully automated robotic platform for chemical synthesis, designed to enable the discovery, synthesis, and catalytic characterization of nanoparticle (NP) heterostructures using a chemical reduction approach. Through a one-step, SDS-mediated synthesis under alkaline conditions, where the surfactant sodium dodecyl sulfate (SDS) stabilizes particle growth, and the alkaline medium promotes oxide formation, we aimed to discover a range of metal oxide semiconductors and their combinations with noble metals for catalysis.^32,33^ We successfully synthesized a range of nanomaterials, including monometallic nanoparticles such as gold nanoparticles (Au NPs), silver oxide nanoparticles (Ag_2_O NPs), and copper oxide nanoparticles (CuO NPs), as well as composite nanomaterials like CuO-Au NP heterostructures and CuO-Ag_2_O NP heterostructures with good reproducibility. In contrast, the other targeted metal oxides (*e.g.,* TiO_2_, NiO) did not produce reproducible products under the same conditions.

For a proof-of-concept model system, we investigated the photocatalytic degradation of the dye Methyl Green (MG). Compared to CuO NPs, the formation of high-quality CuO-Au and CuO-Ag_2_O heterostructures demonstrated enhanced visible light absorption, improving their photocatalytic efficiency in the decomposition of the dye MG. This was validated through real-time spectroscopic monitoring, confirming that the robotic platform can evaluate the catalytic activity of solid CuO-based nanomaterials in the degradation of organic molecules. While we attempted to monitor the self-assembly mechanism in-line during synthesis, instrumental limitations made this approach unfeasible. Instead, off-platform kinetic assays were performed, which revealed the formation times of individual nanoparticles within each heterostructure and allowed us to propose a potential self-assembly mechanism.

Three key steps were performed during the discovery and application of NPs (Figure 1): (1) the synthesis of metal oxide NPs guided by χDL script; (2) characterization of the obtained nanostructures using off-line X-ray diffraction (XRD) and electron microscopy techniques to ensure homogeneity and reproducibility, and (3) investigation of the catalytic activity evaluation of the obtained NPs using in-line UV–vis spectroscopy.

**Figure 1:**
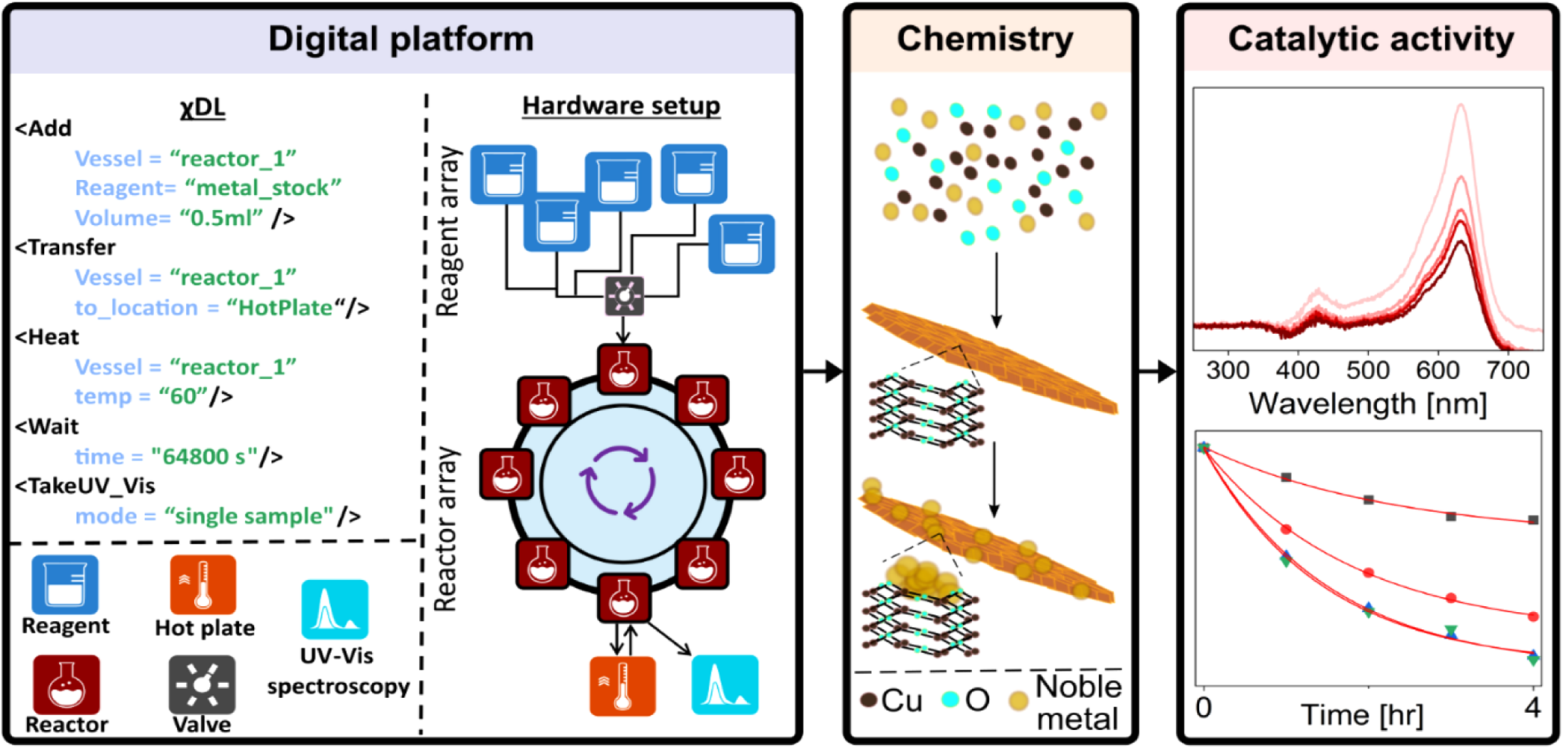
An overview of the workflow is outlined in this paper. The integration of a digital chemical robotics platform for nanomaterial discovery, synthesis, and catalytic performance evaluation of CuO-based NPs. **Left**: the modular automated platform mediated by χDL. **Middle**: The CuO-based NP chemistry. **Right**: the catalytic performance of the NPs with the dye Methyl Green, measured in-line.

## Result and Discussion

### Digital Robotic Platform for Nanoparticle Synthesis

The operation of the robotic platform relies on implementing the synthesis method through a language that expresses synthetic procedures using terminology similar to that found in the literature. These procedures can be executed on any compatible robotic platform. In this work, we employed χDL, which was developed to represent synthetic workflows as sequences of processes occurring in abstract vessels with abstract hardware. The robotic platform facilitates the production of a wide range of solid nanomaterials, from monometallic to composite nanostructures, by mixing up to nine stock solutions, including metal ions, stabilizers, and reducing agents, into designated vials. The core robotic hardware includes a chemical reaction module capable of executing up to 24 syntheses (per Geneva wheel), enabling liquid handling, mixing, heating, sample transfer, and in-line spectroscopic analysis with high precision. Each synthesis process is encoded in χDL steps, allowing the controlled addition of multiple stock solutions. The module utilizes a Geneva wheel mechanism, synchronized with parallel or sequential liquid dispensing, pumps, and reagent stirring, ensuring efficient, precise, and reproducible synthesis processes.^2^ The chemical reaction modules platform is shown in Figure 2 (additional relevant information, including the robotic platform setup, modules, and the χDL steps, is available in Section S1).

**Figure 2:**
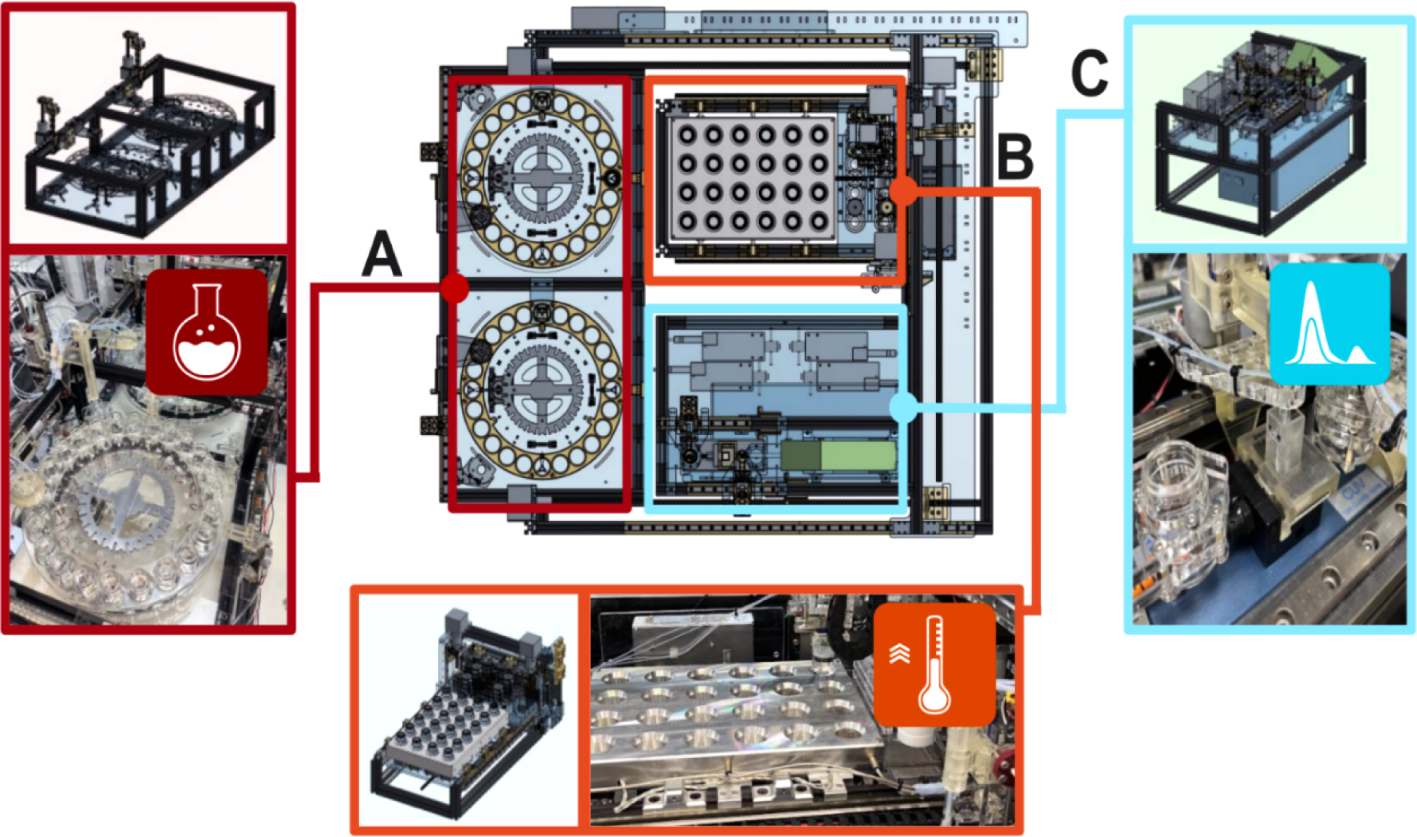
An overview of the robotic platform consists of multiple functional stations, including: (A) two Geneva wheels to initiate the synthesis, (B) a hot plate matrix, and (C) an in-line UV–vis spectroscopy module for real-time analysis.

The stock solution library for the CuO-based NPs contained: copper acetate monohydrate (0.1 M), silver nitrate (10 mM), and chloroauric acid (10 mM) as the source of Cu, Ag, and Au ions, respectively, along with SDS (0.6 M) and NaOH (1 M). We used the following procedure to discover nanomaterials on the robotic platform: (1) Specified quantities of the starting materials were transferred from the stock solutions library into vials on the Geneva wheel under constant stirring. Each sample contained a metal ion stock solution (one or a combination of two), SDS, NaOH, and distilled water to bring the total volume to 10 mL, all dispensed using a liquid handling backbone. (2) Next, each vial was transferred to the heating matrix for 18 h at 70 °C, before being transferred back to the wheel station for cooling to room temperature. (3) To determine the catalytic activity, 10 mg/L of the dye Methyl Green (MG) was added to each sample containing CuO-based nanoparticles. The mixture was stirred, and an aliquot was transferred to a cuvette, where real-time UV–vis spectroscopic feedback was collected every 15 min for 4 h per sample (the χDL steps for designing an experiment are available in the Section S2)

### Reproducibility and Validation

We built the programmable platform to enable high-throughput discovery of composite metal oxide nanoparticles in the solid-phase by setting up the robotic system to mix different metal salt combinations and to evaluate the influence of SDS-mediated surfactant effects under alkaline conditions for Cu, Ti, and Ni, both alone and with noble metals (e.g., Au, Ag). First, we wanted to determine whether the high-throughput platform could reliably synthesize the target NPs in automation. To do this, several samples produced on the platform were compared to samples produced manually across four key parameters: appearance, crystal structure, morphology, and homogeneity. As shown in Tables S1–S3, all CuO-based samples exhibited strong similarity in each parameter, confirming the platform’s high reproducibility without manual intervention. However, TiO_2_-based NPs (Table S4) and NiO-based NPs (Table S5) did not produce consistent results. Next, before applying real-time feedback for catalytic activity, we validated the in-line UV–vis absorbance spectra against off-platform measurements. Silver NP seeds with a distinct plasmonic peak were prepared, followed by gradual addition of gold ions to track the resulting absorbance shifts.^34,35^ As shown in Figure S11, the absorbance spectra were consistent across both instruments, confirming the accuracy of the in-line measurements.

### SDS-Mediated CuO-Based Nanoparticles

Control samples consisting of the monometallic structures were prepared by mixing the parent stock solutions of each metal ion individually with SDS and NaOH solutions. When copper acetate was used, a brown solid was formed in the monoclinic crystal phase with lattice parameters of a = 4.69Å, b = 3.44Å, c = 5.14Å, and angles α = γ = 90°, β ≠ 90° which corresponded to CuO; the solid particles had an average length of 439 ± 70 nm and a width of 171 ± 39 nm (Table S6 and Figure S12A). When chloroauric acid was used, a pink suspension of spherical gold NPs was observed with an average diameter of 34 ± 5 nm (Figure S12B), while the use of silver nitrate resulted in a thin black precipitate, forming small dot-like Ag_2_O NPs with an average size of ∼5 nm (Figure S12C).

Next, we investigated the effect of combining copper ions with gold or silver ions while maintaining the same SDS and NaOH conditions. Upon introducing both metal species into the synthesis, we observed the formation of a uniform nanocomplex comprising two distinct crystal structures:

(1) Cu^2+^ and Au^3+^ coprecipitated to form a CuO-Au NP heterostructure (Figure 3A). Powder XRD analysis confirmed the presence of two attached crystal systems: a monoclinic phase with lattice parameters of a = 4.69Å, b = 3.44Å, c = 5.14Å, and angles α = γ = 90°, β ≠ 90°; and a cubic phase with lattice constants a = b = c = 4.07 Å and angles α = β = γ = 90° (Figure 3C and Table S7). Scanning electron microscopy (SEM) images and energy-dispersive X-ray spectroscopy (EDX), including atomic mapping analysis, confirmed that the nanostructure core corresponded to CuO, while the spherical nanoparticles were identified as gold (Figure S13). High-resolution TEM (HRTEM) further revealed the precise localization of AuNPs at the tips of the CuO surfaces (Figure S14). During the hybridization process, a size reduction was observed in both nanostructures; the CuO nanoleaf exhibited an average length of 231 ± 42 nm and a width of 46 ± 13 nm, with its morphology transforming from a nanoleaf into a toothpick-like shape. The attached AuNPs had an average diameter of 12 ± 6 nm, and ICP analysis indicated that the molar ratio Cu:Au was 211:1 (Table S9).
(2) Cu^2+^ and Ag^+^ coprecipitated to form a CuO-Ag_2_O NP heterostructure (Figure 3B). Powder XRD analysis confirmed the attachment of two distinct crystal systems: a monoclinic phase with lattice constants a = 4.73Å, b = 3.47Å, c = 5.19Å, and angles α = γ = 90°, β ≠ 90°; and a cubic phase with lattice constants a = b = c = 4.77Å and angles α = β = γ = 90° (Figure 3C and Table S8). SEM images and EDX, including atomic mapping analysis, confirmed that the core nanostructure corresponded to CuO, while the spherical nanoparticles contained Ag and O atoms, corresponding to the silver oxide (Ag_2_O) structure (Figure S15). High-resolution TEM further revealed the precise localization of Ag_2_O NPs distributed along the CuO surface (Figure S16). During the hybridization process, a size reduction in the CuO nanostructure was observed; the CuO nanoleaf exhibited an average length of 300 ± 58 nm and a width of 59 ± 17 nm, with its morphology transforming from a nanoleaf to a toothpick-like shape. The attached Ag_2_O nanoparticles had an average diameter of 7 ± 2 nm, and ICP analysis indicated that the molar ratio Cu:Ag was 428:1 (Table S9).

**Figure 3:**
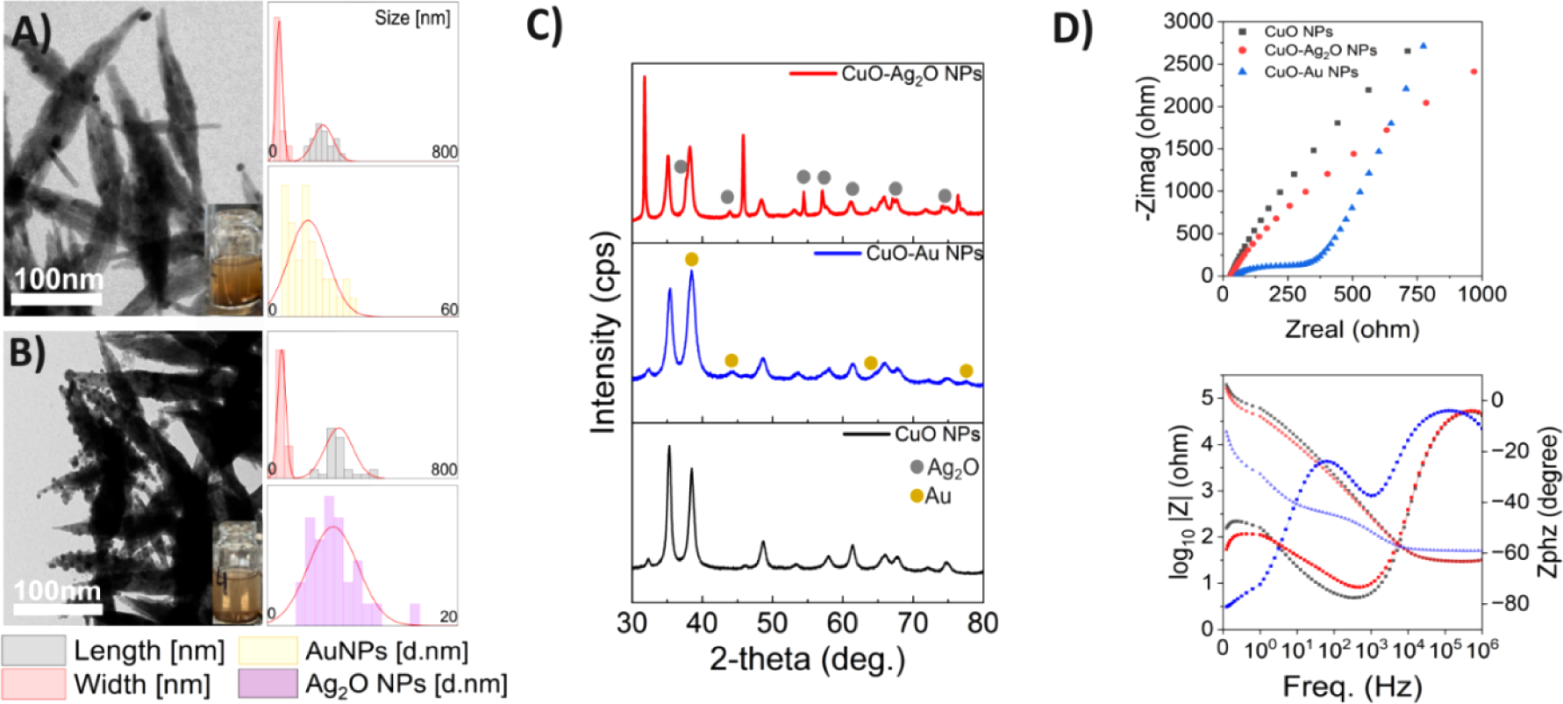
Structural and electrochemical characterization of CuO-based NPs. (A) TEM image of CuO-Au NP heterostructures with corresponding particle size distributions for CuO nanoleaves (length and width) and Au NPs. (B) TEM image of CuO-Ag_2_O NP heterostructures with corresponding particle size distributions for CuO nanoleaves (length and width) and Ag_2_O NPs (C) XRD patterns confirming the monoclinic CuO phase in all samples, with additional reflections corresponding to metallic Au (middle) and Ag_2_O (top). (D) EIS analysis: Nyquist plots (top) showing reduced charge transfer resistance for CuO-Au and CuO-Ag_2_O relative to bare CuO, and Bode plots (bottom) showing lower impedance and enhanced phase response for the heterostructures, consistent with improved charge transport.

For both NP heterostructures, Electrochemical Impedance Spectroscopy (EIS) was performed to evaluate the charge-transfer dynamics (Figure 3D). The Nyquist plots (top) revealed a decrease in charge transfer resistance (R_ct_) for the CuO-Au heterostructures compared to both CuO-Ag_2_O and bare CuO NPs, exhibiting significantly enhanced interfacial conductivity. Similarly, the Bode plots (bottom) indicate lower impedance and higher phase angle shifts for the heterostructures, particularly CuO-Au, consistent with improved charge carrier lifetime and reduced electron–hole recombination.^36^ These results indicate that coupling CuO with noble metals facilitates more efficient charge transport.

Compared to the monometallic phase, the two NP heterostructures exhibited distinct differences in morphology, appearance, and particle size. Both NP heterostructures exhibited a brown solid appearance, with CuO nanoleaf as the core material, coated by spherical nanoparticles. This coating effect led to a significant decrease in CuO nanoleaf size, indicating structural modification upon hybridization. The monometallic CuO nanoleaf had an average length of 439 ± 70 nm and a width of 171 ± 39 nm. However, upon hybridization, CuO-Au NPs showed a 47% decrease in CuO length (231 ± 41 nm) and a 73% decrease in CuO width (46 ± 13 nm), while the complex CuO-Ag_2_O NPs exhibited a 32% decrease in CuO length (300 ± 58 nm) and a 65% decrease in CuO width (59 ± 17 nm). Additionally, the spherical AuNPs attached to the CuO nanoleaves showed a 67% decrease in size, from 34 ± 5 nm (without the presence of Cu ions) to 11 ± 5 nm (with the presence of Cu ions), whereas the Ag_2_O NPs maintained approximately the same size (∼5 nm) in both cases. We observed that AuNPs preferentially aggregated on the tips of the CuO nanoleaves, whereas Ag_2_O NPs were more evenly distributed along the CuO surface. The observed size decrease of each nanoparticle within the nanocrystal suggests that in a one-step synthesis method involving two metal ions and limited precursors (SDS and NaOH), the nucleation and growth of each NP is constrained. This limitation likely arises due to competition for available reactants and the simultaneous formation of multiple phases within the same reaction environment. The noble metal ions (Au, Ag) appear to preferentially attach to the CuO surface, serving as anchoring sites for their subsequent growth into plasmonic NPs.

### Real-Time Spectroscopic Feedback for Photodegradation Activity

To evaluate the catalytic performance of the synthesized CuO-based NPs, we monitored the real-time photodegradation of the dye Methyl Green (MG). We selected MG as a proof-of-concept model for exploring real-time spectroscopic feedback in the robotic platform due to its well-known photodegradation properties, simple visible color change, and the ability to monitor catalytic performance via UV–vis spectroscopy, making it suitable for the study.^37^ By comparing the degradation rate of CuO-metal NPs to that of CuO alone, we aimed to demonstrate the effect of incorporating metals into the CuO structure, highlighting improvements in the degradation rate due to the synergistic effects of the hybrid material with noble metals and the increased CuO surface area resulting from size reduction. For the in-line study, the spectroscopic measurements were conducted in two operational modes: “reference” and “multiple samples.” In the “reference mode,” the spectrometer recorded a raw spectrum as a baseline, whereas in the “multiple samples mode,” spectra were collected at defined intervals and converted into real-time absorption intensity graphs (Figure S10). For fully automated real-time spectroscopic feedback, we utilized a UV–vis XZ liquid handler equipped with two dedicated tubes: one for injecting water to clean the UV–vis cell before and after each experiment, and one for transferring the measured sample from the wheel station (Figure 4A).

**Figure 4:**
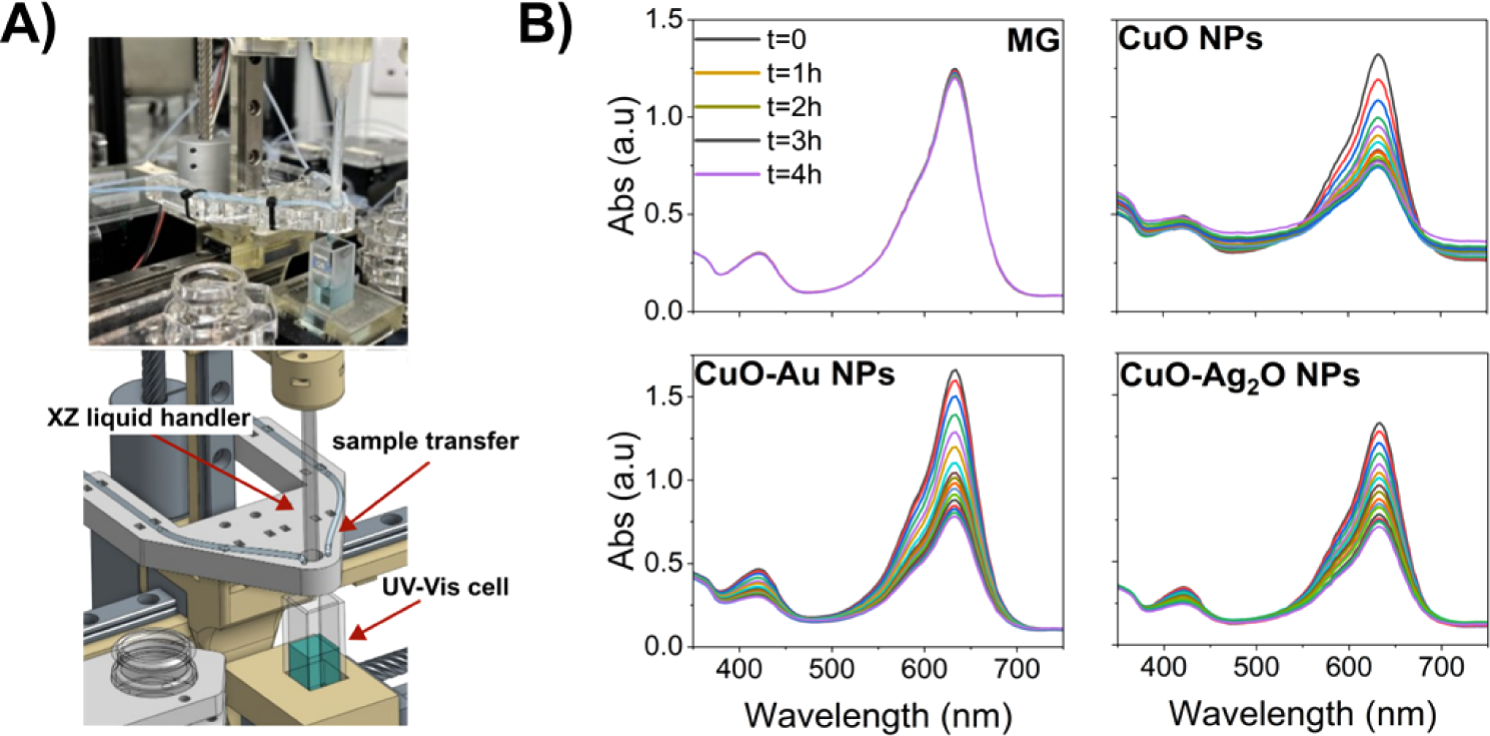
Real-time spectroscopic monitoring of photocatalytic activity. A) An image and schematic representation of the in-line UV–vis spectroscopy station integrated into the robotic platform; B) Time-dependent UV–vis absorbance spectra recorded over 4 h for photodegradation of Methyl Green (MG) in the presence of three photocatalysts: CuO, CuO-Au, and CuO-Ag_2_O.

In each experiment, 5 mg of each photocatalyst (CuO, CuO-Au, or CuO-Ag_2_O) was manually weighed and placed back into a separate vial. Afterward, all subsequent steps were performed automatically: (1) The photocatalyst was dispersed in 12 mL of a 10 mg/L aqueous MG solution under continuous stirring. (2) 1.5 mL of the reaction mixture was transferred from the vial to the UV–vis cell for absorbance measurements at 15 min intervals over a total duration of 4 h. (3) The collected absorbance spectra provided time-resolved insights into the degradation kinetics of MG in the presence of each catalyst.

The in-line spectroscopic feedback system successfully captured the degradation profiles of MG under different photocatalytic conditions, including MG alone, MG with CuO NPs, MG with CuO-Au NPs, and MG with CuO-Ag_2_O NPs. The UV–vis spectroscopic data confirmed a progressive decrease in dye absorbance over time under constant light exposure, indicating successful photodegradation. The results demonstrated a direct correlation between exposure time and dye degradation, with the percentage degradation increasing as the reaction progressed. In a control experiment without photocatalysts (MG only), we observed a spontaneous degradation of 11 ± 6% over 4 h. The pristine CuO NPs exhibited 45 ± 2% degradation ability, whereas the CuO-Au and CuO-Ag_2_O heterostructures showed enhanced degradation efficiencies of 57 ± 3% and 65 ± 2%, respectively, as shown in Figure 4B. To determine whether the observed photocatalytic enhancement originated solely from noble-metal activity independent of the CuO host, we evaluated the catalytic performance of unsupported Au NPs and Ag_2_O. The measurements were carried out with <1 mg of noble metal, approximating the loading present in the CuO-based NPs as confirmed by ICP analysis. As shown in Figure S17, only minimal dye degradation was observed for the pure noble metals (5–12%). These results indicate that the enhanced photocatalytic activity of the CuO-based NPs arises not only from the noble metals alone but from their synergistic coupling with the CuO host. Therefore, we assume that interaction with noble metals reduced the size of the CuO host, increased its surface area, and created additional active sites for dye adsorption. Their attachment to the CuO surface also showed an additional surface plasmon resonance (SPR) effect, facilitating charge transfer. Even without noble metal attachment, CuO alone remained dominant, achieving nearly 50% photodegradation. This intrinsic activity is attributed to the semiconductor properties of CuO, which enable visible-light absorption, generation of electron–hole pairs, and subsequent redox reactions driving dye degradation.^19,21,38^

### Proposed Mechanism and Self-Assembly

We observed a compelling relationship between the nanostructures during the hybridization process. Structural characterization revealed that mixing Cu ions with noble metal ions (*e.g*., Au, Ag) induced a morphological transformation from nanoleaf to nanotoothpick, accompanied by a noticeable decrease in size (Figure 5A) when these metal ions were used. To gain deeper insights into the nucleation dynamics of NP heterostructures, where metal ions accumulate into a new phase on the CuO surface for crystal growth, we conducted real-time monitoring using off-platform measurements, as *in situ* monitoring at the optimal temperature was not feasible in the robotic platform. In addition to this challenge, due to analytical constraints, we were only able to reach a maximum temperature of 42 °C, rather than the intended 70 °C, at which the synthesis was typically performed. Despite this, the kinetic profile obtained still provided valuable insights into the formation sequence of the CuO-based NP heterostructure, enabling the proposal of a self-assembly and self-organization mechanism.

**Figure 5:**
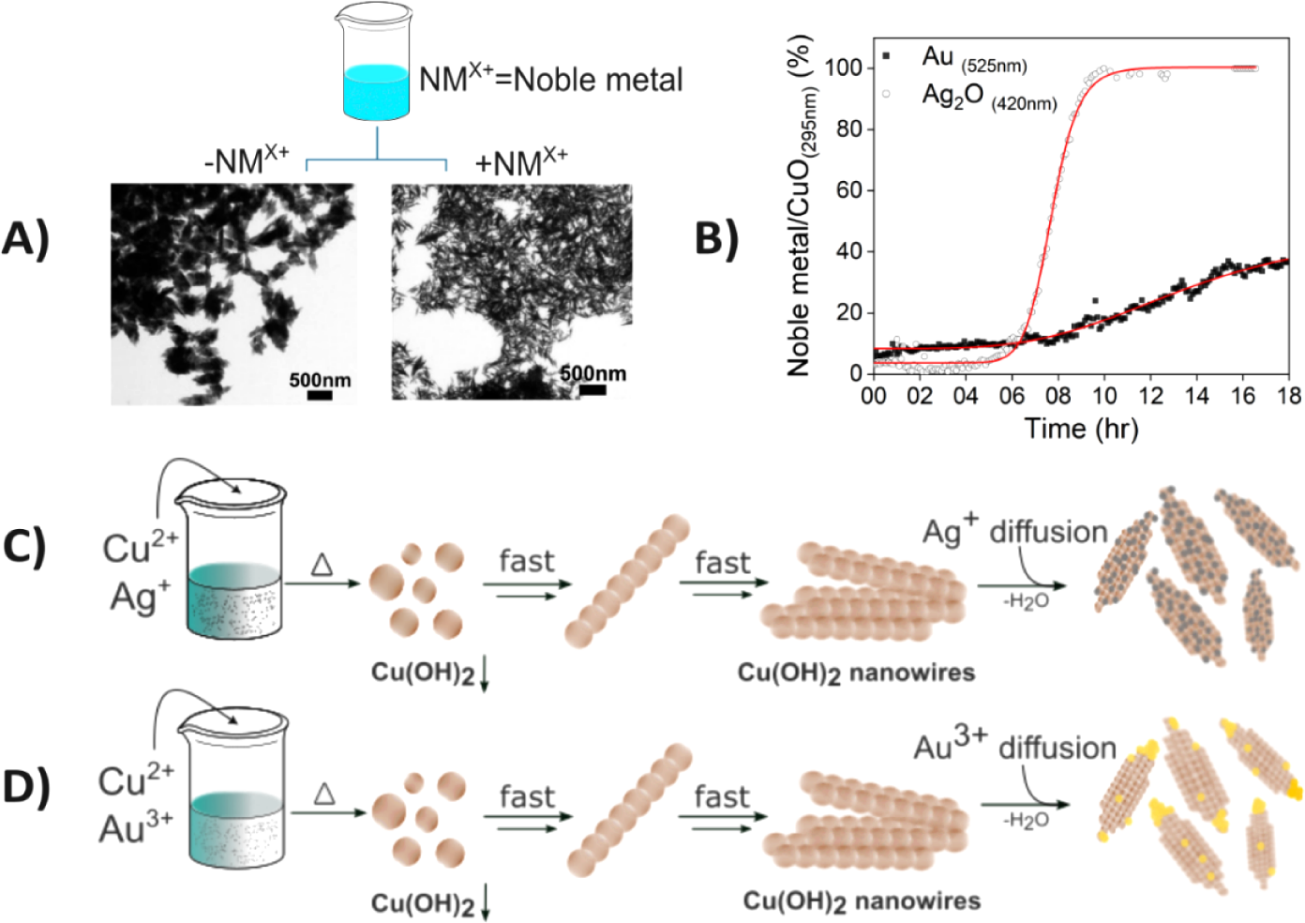
CuO-based self-assembly and proposed mechanism. A) SEM images comparing CuO nanoleaf structures w/o decorated with noble metal ions (NM^X+^ = noble metal ions); B) Kinetic assay tracking the formation of CuO-Au and CuO-Ag_2_O NP heterostructures over time, showing time-dependent nucleation and growth; C) Schematic illustration of the proposed self-assembly mechanism of CuO-Au NP heterostructures, highlighting the nucleation and attachment of gold NPs on the tips; D) Schematic illustration of the proposed self-assembly mechanism of CuO-Ag_2_O NP heterostructures, highlighting the nucleation and attachment of silver oxide NPs along the surface.

The formation of each NP heterostructure was tracked over time using UV–vis spectroscopy, enabled by the distinct absorption features of gold (Au) and silver oxide (Ag_2_O) exhibit distinct absorption spectra, with maximum absorption peaks at 520–530 nm and 420–430 nm, respectively.^39,40^ To track the formation of each NP heterostructure over time, we monitored the absorbance changes for CuO-Au and CuO-Ag_2_O at 525 and 420 nm, respectively. As shown in Figure 5B, the CuO-Au NP heterostructure showed no significant change in absorbance at 525 nm during the first 8 h, whereas the CuO-Ag_2_O NP heterostructure exhibited little change in absorbance at 420 nm for the first 5 h. Subsequently, AuNPs exhibited a slow, gradual increase in intensity over the next 10 h, while Ag_2_O NPs showed a dramatic increase over the next 5 h, reaching saturation by the end of the assay. This time-dependent absorbance behavior suggests a common two-step formation mechanism: (1) The absence of absorbance changes in the early hours indicates that CuO nanoleaves are being crystallized first, providing a structural platform as a core material. (2) Once CuO NP is fully formed, noble metal ions (Au^3+^, Ag^+^) begin to accumulate on the CuO surface and grow into spherical plasmonic NPs in a logistic trend. Additionally, in all Cu-containing experiments, we observed a color change from blue to brown within the first few hours, further supporting the kinetic findings and confirming CuO formation.

It was seen that in the presence of an alkaline environment (pH ∼ 11), the high concentration of OH^–^ ions accelerates the formation of the initial transparent blue [Cu­(OH)_4_][Bibr ref2]– complex, which thermally decomposes to precipitate Cu­(OH)_2_ as nanowires, which are insoluble in the aqueous solution.^18,41^ The final conversion from Cu­(OH)_2_ to CuO in solution was found to occur through a transformation which involves a dehydration process and the formation of an oxo-bridge (-O−) between two Cu atoms to form the O–Cu–O bridges and aggregate into the entire structure of CuO nanoleaf.^41–43^ However, when Cu ions interact with either gold or silver ions in the same reaction environment, we observed significant morphological transformations in the CuO nanoleaf size distribution. The CuO nanoleaf surface area decreased by ∼ 70%, indicating a disruption of its initial growth driven by the adsorption of noble metal ions. The observed changes indicate that Cu, Au, and Ag ions compete for precursor consumption (i.e., NaOH and SDS) to form the most thermodynamically stable structure. This competition for limited precursors creates a dynamic interaction between individual NPs in the NP heterostructure self-assembly, which we explore in this section.

This behavior can be attributed to a competition between metal ions driven by distinct reaction pathways, thermodynamic versus kinetic control. Strongly alkaline conditions promote rapid hydrolysis and condensation of copper ions, leading to accelerated CuO nucleation. These conditions also influence the redox behavior of metal ions by shifting their reduction potentials; while noble metal ions are generally more stable in basic environments, silver ions are reduced faster than gold ions.^44^ However, their reaction with hydroxide is relatively slower and does not lead to precipitation as readily as in the case of copper ions, which immediately form Cu­(OH)_2_ under similar conditions.^44–46^ Silver ions react with hydroxide to form an unstable intermediate silver hydroxide (AgOH), which quickly dehydrates to silver oxide (Ag_2_O), leading to visible precipitation.^47^ In the case of gold chloride, the hydroxide ions gradually replace chloride ligands to form the more stable intermediate complex [AuCl_4‑X_(OH)_X_^X–^], which displays diminished reactivity and therefore slows nanoparticle formation.^44,48^ The presence of CuO facilitates the reduction of the complex to Au^0^ by enabling surface-mediated electron transfer and supporting the localized growth of gold nanoparticles. The presence of hydroxyl (−OH) groups and oxygen-rich sites on the CuO surface, combined with the AgOH relative to the more stable [AuCl_4‑X_(OH)_X_^X–^] complex, promotes more efficient and uniform adsorption of silver ions. This leads to the rapid formation of Ag_2_O NPs, in contrast to the slower and more localized nucleation of Au NPs, as shown in Figure 5B.^44,49–52^

Interestingly, the Ag content determined by EDX (<0.1 wt %) was consistent with the ICP-OES data, suggesting a more homogeneous distribution of Ag_2_O within the CuO-Ag_2_O heterostructure. In contrast, the CuO-Au sample exhibited a significant difference between surface composition (EDX: ∼ 4.0 wt %) and bulk content (ICP-OES: 0.06 wt %), indicating enrichment of Au domains. According to images, AuNPs were found to preferentially aggregate at the tips of the CuO nanoleaves, whereas Ag_2_O nanoparticles appeared to distribute more uniformly along the CuO surface. The tips of CuO nanoleaves have higher surface energy compared to the interior CuO surface due to defects and uncoordinated atoms that increase the density of active sites. Au ions have a strong preference for high-energy sites with a higher density of reactive oxygen species, which supports their interaction with gold ions, leading to the formation of metallic gold.^53,54^ We assume that the tips of the CuO nanostructures may serve as localized reduction centers, where the gold complex receives electrons and undergoes nucleation. These regions likely favor the initial formation and growth of AuNPs due to enhanced electron transfer and surface reactivity (Figure 5C). In contrast, silver ions are more reactive and less noble than gold, making them less likely to form selective interactions with high-energy tip sites. The CuO surface provides a stable environment with fewer specific high-energy sites, leading Ag_2_O NPs to grow more homogeneously and less locally than AuNPs (Figure 5D).^55,56^ Another important aspect is that, under the thermal conditions, the CuO, as a semiconductor, can facilitate surface-mediated electron transfer to the metal ions, promoting their reduction and deposition onto the CuO surface.^57,58^ Therefore, by accepting electrons, the noble metal ions are reduced and deposited as nanoparticles. Once deposited onto the CuO surface, these nanoparticles are assumed to undergo aggregation via Ostwald ripening- a thermodynamically driven process in which larger particles grow at the expense of smaller ones to minimize the overall surface energy.^59^ This process plays a crucial role in shaping the final NP heterostructures by influencing their size, morphology, and overall structural formation.

## Conclusion

We successfully developed an automated chemical robotic platform for the discovery, synthesis, and catalytic characterization of solid nanomaterials. This platform integrates a fully digitized workflow based on χDL steps, enabling reproducible, rapid, and high-throughput synthesis of CuO-based semiconductor materials, including CuO-Au and CuO-Ag_2_O NP heterostructures. The photocatalytic degradation of Methyl Green was monitored in real time using spectroscopic feedback, serving as a proof of concept for solid-phase activity. The results indicate that the majority of the degradation arises from the intrinsic semiconductor properties of CuO, with the SPR effect from the attached noble metals providing a minor contribution.

To further investigate the dynamic between CuO and noble metal NPs during the hybridization process, we conducted off-platform kinetic assays to track the nucleation and growth of each NP heterostructure. Unfortunately, these assays could not be fully automated due to instrumental limitations, requiring off-line measurements. Despite this constraint, we observed that CuO crystallized first, followed by the nucleation and attachment of noble metal ions, leading to morphological transformations from nanoleaf to nanotoothpick structures. Interestingly, AuNPs mostly aggregated at the tips of the CuO nanoleaf, whereas Ag_2_O NPs were more uniformly distributed along the CuO surface. Based on these observations, we proposed that the hybridization process is driven by competition for precursor consumption, where Cu, Au, and Ag compete for NaOH and SDS to achieve the most thermodynamically stable NP. Furthermore, impedance spectroscopy over a range of frequencies confirmed not only the successful attachment of the noble metals to the CuO surface but also enhanced conductivity, with increased charge transfer relative to CuO alone.

Overall, this study highlights the successful integration of digital chemistry into solid-phase materials discovery, marking a significant shift from labor-intensive manual experimentation to a programmable, automated workflow. This approach improves efficiency by reducing synthesis time and cost, ensuring consistent reproducibility, and moving beyond colloidal, plasmonic-focused systems. However, a major challenge remains in enabling real-time exploration of self-assembly processes, as the complex dynamics of nucleation and growth require adaptive feedback control. Future work should address these limitations by refining automation strategies, broadening the library of advanced nanostructures, extending catalytic studies to additional pollutants such as pharmaceuticals and pesticides, and integrating machine learning to predict and optimize reaction pathways in real time.

## Experimental Section

### Materials

All stock solutions used Type I ultrapure water (18.4 MΩ•cm). Copper­(II) acetate monohydrate (Cu­(CO_2_CH_3_)_2_·H_2_O, ≥ 98%, Sigma-Aldrich), Silver nitrate (AgNO_3_, 99.9%, Sigma-Aldrich), Gold­(III) chloride trihydrate (HAuCl_4_, 99.9%, Sigma-Aldrich), Dodecyl sulfate sodium (SDS) salt (C_12_H_25_NaO_4_S, Sigma-Aldrich), Sodium hydroxide (NaOH, 98%–100.5%, Honeywell Fluka), absolute ethanol (C_2_H_6_O, > 99.70%).

### Synthesis of CuO NPs

0.5 mL of 0.1 M aqueous copper­(II) acetate monohydrate solution was mixed with 0.5 mL of 0.6 M aqueous sodium dodecyl sulfate (SDS) solution. To this mixture, 0.18 mL of 1 M aqueous NaOH and 8.82 mL of distilled water were added under stirring. The resulting solution was stirred for 5 min at room temperature, then heated to 70 °C and maintained at that temperature for 18 h. The as-prepared sample was centrifuged and thoroughly washed with distilled water and ethanol three times to form the CuO nanoleaf as a brown precipitate.

### Synthesis of Au NPs

0.5 mL of 10 mM aqueous chloroauric acid solution was mixed with 0.5 mL of 0.6 M aqueous sodium dodecyl sulfate (SDS) solution. To this mixture, 0.18 mL of 1 M aqueous NaOH and 8.82 mL of distilled water were added under stirring. The resulting solution was stirred for 5 min at room temperature, then heated to 70 °C and maintained at that temperature for 18 h, to form a pink suspension of spherical gold NPs.

### Synthesis of Ag_2_O NPs

0.5 mL of 10 mM aqueous silver nitrite solution was mixed with 0.5 mL of 0.6 M aqueous sodium dodecyl sulfate (SDS) solution. To this mixture, 0.18 mL of 1 M aqueous NaOH and 8.82 mL of distilled water were added under stirring. The resulting solution was stirred for 5 min at room temperature, then heated to 70 °C and maintained at that temperature for 18 h. The as-prepared sample was centrifuged and thoroughly washed with distilled water and ethanol three times to form a black precipitate of spherical Ag_2_O NPs.

### Synthesis of CuO-Based NP Heterostructures

0.5 mL of 0.1 M aqueous copper­(II) acetate monohydrate solution was mixed with 0.5 mL of 10 mM aqueous noble metal salt solution, either gold ions (for CuO-Au NPs) or silver ions (for CuO-Ag_2_O NPs). Next, 0.5 mL of 0.6 M aqueous sodium dodecyl sulfate (SDS) solution was added. To this mixture, 0.18 mL of 1 M aqueous NaOH and 8.32 mL of distilled water were added under stirring. The resulting solution was stirred for 5 min at room temperature, then heated to 70 °C and maintained at that temperature for 18 h. The as-prepared sample was centrifuged and thoroughly washed with distilled water and ethanol three times to form a brown precipitate of CuO-based NP.

### Characterization

The crystal structure of the nanoparticles was analyzed by X-ray powder diffractometer (XRD) of Rigaku Miniflex X-ray diffractometer 300/600 with an X-ray generator (40 kV, 15 mA) at a scanning rate of 0.80° min^–1^ from 3° to 80°. Transmission electron microscope (TEM) images were obtained by a JEOL 1400 FLASH TEM running at 80 kV. The tif/jpeg Images were captured using TEM Center software version 1.7.26.3016, and an inbuilt JEOL FLASH CCD camera. Ultra High-Resolution Scanning Electron Microscope (UHR-SEM) images were obtained by TESCAN CLARA with a Field Emission Gun electron source that allows users to examine the microstructure, morphology, and surface characteristics in high-resolution and low-energy imaging. Energy Dispersive Spectrometry (EDS) analysis was obtained by Oxford Instruments UltimMax 65 with Aztec live interface. Ultraviolet–visible spectrophotometry (UV–vis) absorbance was measured in the visible range 400–1000 nm with an in-line UV–vis spectroscopy from Ocean Optics with their Python package. Thermaogravity analysis (TGA) was performed on a TA Instruments Q 500 Thermogravimetric analyzer under air flow with a heating rate of 10 °C min^–1^ up to 1000 °C. Samples were heated in an LTE OP60-UF oven equipped with fan circulation. Elemental analysis for Cu, Au, and Ag was performed on a Leeman inductivity-coupled plasma (ICP) spectrometer. Electrochemical impedance measurements (EIS) were recorded using Gamry Instruments software. A three-electrode arrangement was used, consisting of the working electrode with an aliquot of the sample (0.071 cm^2^ surface area), a reference electrode (silver/silver chloride), and a carbon rod, in 25 mL aqueous 0.1 M Na_2_SO_4_. The impedance spectra were generated by applying a sinusoidal signal of amplitude 10 mV over the frequency range 0.1 Hz-1 MHz. The resultant spectra were analyzed with Gamry Echem Analyst software.

## Supplementary Material





## Notes

Additional characterization data, including microscopy images and XRD patterns. Validation tests comparing robotic platform synthesis with manual experiments (PDF)
Details of the programmable robotic platform setup, including modules, XDL steps, and experiment design (ZIP)
