# Supplementary material for: Automated Digital Discovery and Synthesis of CuO-Based Nanoparticle Heterostructures for Catalysis
Source: ACS Appl Mater Interfaces. 2025 Oct 11;17(42):58147–56. doi: 10.1021/acsami.5c13709 (PMC12557214; doi:10.1021/acsami.5c13709)
Supplement: Supplementary file 1 [file am5c13709_si_001.pdf]

Supporting information

Automated Digital Discovery and Synthesis of CuO-Based Nanoparticle Heterostructures for Catalysis

Daniel Hervitz, Yibin Jiang, Daniel Salley, Mark McNulty, Philip. J Kitson and Leroy Cronin*

School of Chemistry, The University of Glasgow, University Avenue, Glasgow G12 8QQ, UK.

*Corresponding authors' E-mail: Lee.Cronin@glasgow.ac.uk

Contents

1. The robotic platform 3
  1.1. General knowledge 3
    1.1.1. TriContinent pumps 3
    1.1.2. Arduino 4
    1.1.3. Commanduino 4
    1.1.4. Chembotbridge 4
  1.2. Modules 9
    1.2.1. Wheel station 11
    1.2.2. XZ liquid handler 13
    1.2.3. Dispensing Unit 14
    1.2.4. Hotplate matrix 16
    1.2.5. Capping unit 18
    1.2.6. UV-Vis module 19
    1.2.7. XYZ gripping arm 21
  1.3. XDL steps and basic steps 21
    1.3.1. Add 22
    1.3.2. Transfer 22
    1.3.3. TransferVessel 22
    1.3.4. SealVessel 23
    1.3.5. UnsealVessel 23
    1.3.6. HeatChillToTemp 23
    1.3.7. StartStir 23
    1.3.8. StopStir 24
    1.3.9. TakeUVVis 24
2. Design an experiment using XDL 25

2.1. Nanoparticles Synthesis 26
2.2. Photocatalytic Activity assay 27
3. Characterization 28
3.1. Validation: CuO-based NPs synthesis 28
3.2. Trials: TiO_2-based NPs synthesis 31
3.3. Trials: NiO-based NPs synthesis 32
3.4. Validation: in-line UV-Vis spectroscopy station 33
3.5. Powder X-ray diffraction – additional data 34
CuO NPs 34
CuO-Au NP heterostructure 36
CuO-Ag_2O NP heterostructure 38
3.6. Electron microscopy images 40
3.6.1. Monometalic NPs - control 40
3.6.2. CuO-Au NP heterostructure 41
3.6.3. CuO-Ag_2O NP heterostructure 43
3.7. Inductively Coupled Plasma Optical Emission Spectroscopy (ICP-OES) 44
3.8. Photodegradation- Control samples 45
4. References 46

1. The robotic platform

1.1. General knowledge

1.1.1. TriContinent pumps

TriContinent pumps were used in the system for fluidic operations and controlled via a Python interface called pycont. This consisted of **four-way distributed valves**, and **sometimes six-way distributed valves**, allowing multiple configurations (see below).

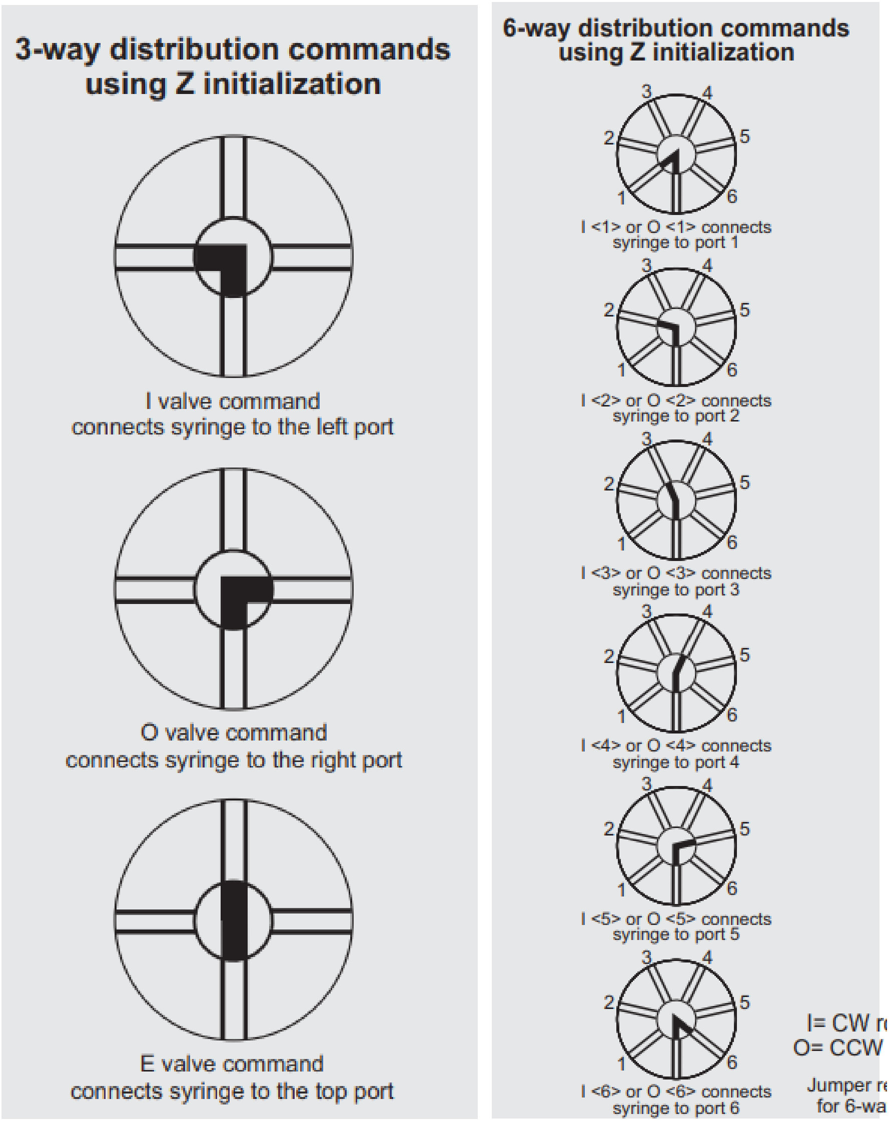

Figure S1: Pump configurations for the four-way distributed valve and the six-way distributed valve.

The figure shows the I/O/E configuration for the four-way distributed valve and the 1/2/3/4/5/6 configuration for the six-way distributed valve. The bottom port is always connected to a syringe. When we want to transfer a liquid using the pump, we will set the configuration of the

pump to select a certain port (I/O/E). The syringe will then actuate, taking liquid from that port. By doing so, the liquid is drawn inside the syringe. Then we will reconfigure the pump so that another port is connected to the syringe. Finally, we move the syringe back and deliver the liquid to that port.

1.1.2. Arduino

Arduino is a microchip we use to control the motion of motors and write analogue signals to the system. We can rely on external libraries for Arduino to have flexible control. But the Arduino website is a good start to get a rough feeling of how it can be used: https://docs.arduino.cc/built-in-examples/.

1.1.3. Commanduino

Commanduino is an external library that we used in the system for two things:

1. Control the motion of motors.
2. Write analogue signals.

A 12V power supply, Arduino MEGA, RAMPS 1.4, NEMA stepper motors, and DVR8825 stepper motor driver board (per motor) are needed to control the stepper motor.

1.1.4. Chembotbridge

By far, we have discussed the usage of pycont to control Tricont pumps, and Commanduino to control stepper motors or write analogue signals.

In principle, we decompose an experimental procedure into the operations of pumps and motors and use pycont and Commanduino to execute the experiments. However, this will make code management difficult, therefore, we created a library called Chembotbridge to control individual modules of the platform.

After installing pycont and Commanduino and making sure the connections are correct, we can assemble the pumps and motors into classes.

To initialize the Chembotbridge, we need to create a configuration file, which records all the necessary hardware information.

The configuration file consists of two important parts: boards and modules, which will be introduced one by one.

Boards

Each board was numbered using "board_1" and so on to prevent conflicting names, as well as defining all the serial communications. They were then categorised as one of four types under "driver":

1. Triconthub (for pumps), where we define the driver type as "tricont". Here is an example of "board_3" with its driver type as "tricont" and baud rate:

```
board_3: # tricont boards around the wheel
  driver: tricont
  io:
    port: COM9
    baudrate: 9600
    timeout: 1
  default:
    volume: 5
    micro_step_mode: 2
    top_velocity: 15000
    initialize_valve_position: I
```

   The example states "board_3" with its driver type as Tricont and baud rate as 9600. If you want to initialize a new board, you need to define all the related properties as shown in the figure. The port of the board can be found via "Device Manager" on a Windows system.

2. Arduino board (for temperature control), where we define the driver type as "temperature_controller":

```
board_6: # the temperature controller of the heating mantle
  driver: temperature_controller
  ios:
    port: COM5
    baudrate: 115200
```

3. Arduino board with RAMS 1.4 (for motor control), where we defined the driver type as "Commanduino":

```
board_1: # wheel left
  driver: commanduino
  ios:
    - port: COM8
```

   This driver type doesn't require the baud rate to be defined.

4. Direct USB communication of the spectrometer, where we define the driver type as "QEPro2192":

```
board_11: # the UV-Vis spectromer
  driver: QEPro2192
```

Nothing additional needs to be defined – the system automatically finds what spectrometers are connected to the computer.

Modules

The second part consists of modules. The modules can require multiple devices, which are controlled by different boards. For example, if we want to have a module that can move in X and Z directions, and be able to dispense liquid, three devices would be required:

1. A motor controlling the motion in the X direction.
2. Another motor controls the motion in the Z direction.
3. A pump (or many pumps) that can do liquid operations.

These motors and pumps are controlled by different boards.

In Chembotbridge, once we define the module, we first need to clarify the class of the module. To define an X/Z liquid handler, we do the following:

1. Define the name of the module as well as its class (Here, the name is XZLiquidHandlerLeft, and the class is XZLiquidHandler):

```
XZLiquidHandlerLeft:
  class: XZLiquidHandler
  devices:
    X_motor: # predefined name···
    Z_motor: # predefined name···
    tricont_pumps:···
```

2. It requires us to fill up the information of X_motor, Z_motor, and Tricont pumps. Such information will be used by Commanduino and tricont to initialize the device. Let's fill up the information for the motors first:

```yaml
XZLiquidHandlerLeft:
  class: XZLiquidHandler
  devices:
    X_motor: # predefined name
      command_id: E1_3
      board: board_1
      config:
        reverted_switch: true
        reverted_direction: false
        enabled_acceleration: false
        speed: 12000
        max_speed: 12000
        homing_speed: 8000
        acceleration: 5000
    Z_motor: # predefined name···
    tricont_pumps:···
```

Take the definition of X_motor as an example.

To define the motor, we need to define the command_id and the board. In the previous example, we initialised board_1 with a type of Commanduino, and it will be used to control the X_motor. Here, when we upload the script, we define the names of the motor as X_3, Y_3, Z_3, E0_3, and E1_3. With the command_id and the board name, we clarify that the **E1_3 motor from board_1** will be used as the X_motor of the XZLiquidHandlerLeft, while XZLiquidHandlerLeft is an object initialised from the class called "XZLiquidHandler". Similarly, the Z_motor is defined by the E0_3 motor from board_1. More information, such as reverted_switch, speed, max_speed, etc., can also be defined for each motor.

The tricont pump information should also be populated. An example is below:

```yaml
XZLiquidHandlerLeft:
  class: XZLiquidHandler
  devices:
    X_motor: # predefined name···
    Z_motor: # predefined name···
    tricont_pumps:
      pump_sample:
        switch: 2
        board: board_3
        top_velocity: 15000
        volume: 12.5
        initialize_valve_position: I
      pump_clean_left:
        switch: B
        board: board_3
        top_velocity: 15000
        volume: 12.5
        initialize_valve_position: I
      pump_waste_left:
        switch: A
        board: board_3
        top_velocity: 15000
        volume: 5
        initialize_valve_position: I
```

This example shows that three pumps (pump_sample, pump_clean_left and pump_waste_left) are used for XZLiquidHandlerLeft. While defining each pump, we need to define five properties:

1. switch:
2. board
3. top_velocity
4. volume
5. initialize_valve_position

The “switch” and “board” clarified which pump we were using uniquely. “Top_velocity” restricts the moving speed of the syringe, and “volume” defines the volume of the syringe. The “initialize_valve_position” is used when the pumps are initialised, which can be I/E/O for four-way distributed valves and 1-6 for six-way valves.

1.2. Modules

In this section, we will introduce the individual modules' structure and software control.

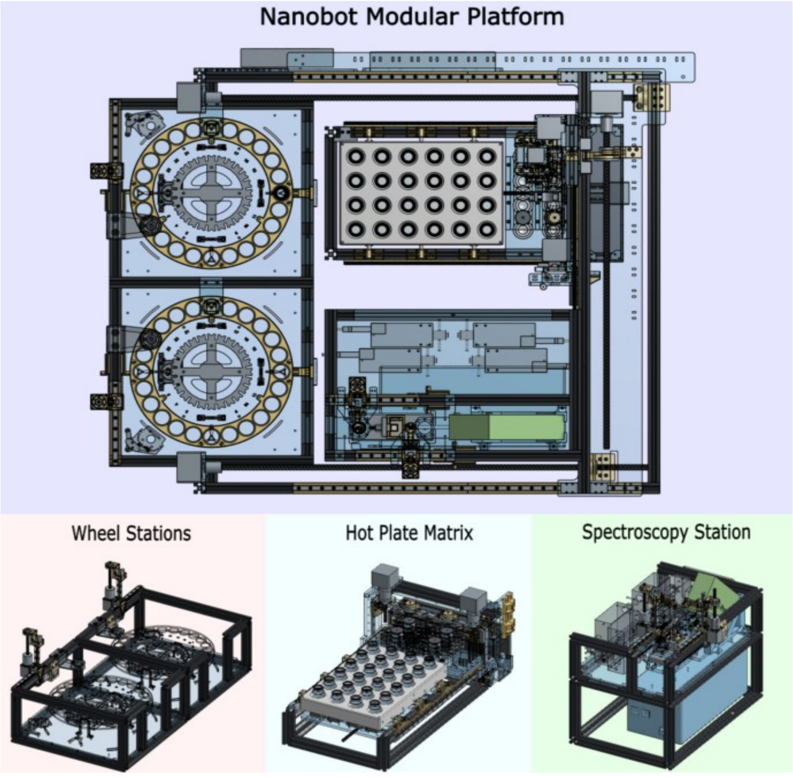

Figure S2. The assembly of the modular platform.

Before we introduce the modules, the definitions of the individual boards are listed below, so the readers can refer to them:

```
ver: 1.0.0
boards:
  board_0: # gripping arm
    driver: commanduino
    ios:
      - port: COM10
  board_1: # wheel left
    driver: commanduino
    ios:
```

```
    - port: COM8
board_2: # wheel right
  driver: commanduino
  ios:
    - port: COM17
board_3: # tricont boards around the wheel
  driver: tricont
  io:
    port: COM9
    baudrate: 9600
    timeout: 1
  default:
    volume: 5
    micro_step_mode: 2
    top_velocity: 15000
    initialize_valve_position: I
board_4: # TiO2 storage syringe driver
  driver: commanduino
  ios:
    - port: COM4
board_5: # control the capping unit for the heating mantle
  driver: commanduino
  ios:
    - port: COM12
board_6: # the temperature controller of the heating mantle
  driver: temperature_controller
  ios:
    port: COM5
    baudrate: 115200
board_7: # controlling uv_vis MSDs
  driver: commanduino
  ios:
    - port: COM6
board_8: # uv_vis to control the light source
```

```
  driver: commanduino
  ios:
    - port: COM11
board_9: # tricont board1 around UV-Vis
  driver: tricont
  io:
    port: COM16
    baudrate: 9600
    timeout: 1
  default:
    volume: 5
    micro_step_mode: 2
    top_velocity: 15000
    initialize_valve_position: I
board_10: # the UV-Vis spectrometer
  driver: QEPro2192
```

1.2.1. Wheel station

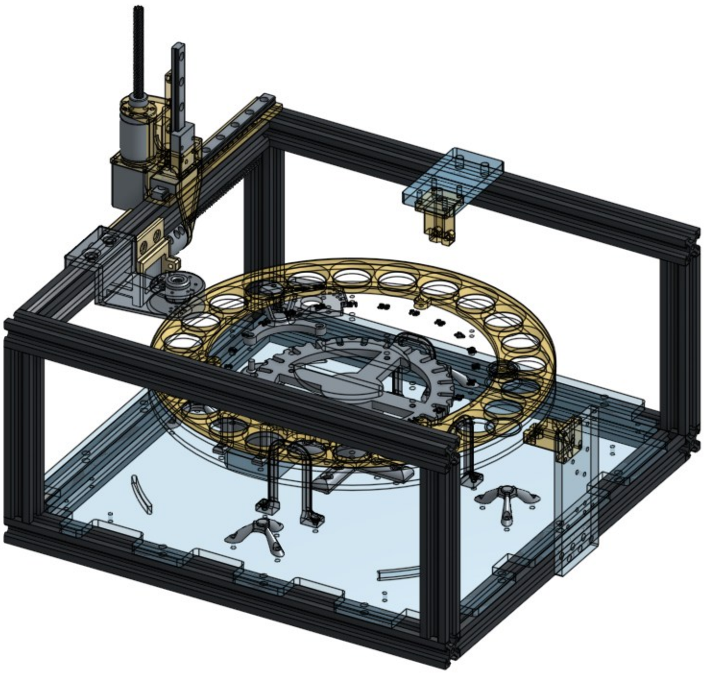

Figure S3: CAD image of the wheel module.

The wheel module uses a 24-vial plate which rotates using a Geneva wheel mechanism, driven by a Nema motor. (http://benbrandt22.github.io/genevaGen/)
At the bottom of the vial plate, we used a combination of magnets attached to fans. When on, the orientation of the magnetic field changes, which rotates the stirring bars in the vial.

In summary, for the wheel, we need to control the following items:

1. A Nema motor for the rotation of the wheel.
2. A port to control the on/off of the stirring fans by writing analogue signals; the speed of the fans can also be controlled by the value of the analogue signal.

Here, you may notice that an X/Z moving unit is not considered part of the wheel. This is because this unit could be mounted without the wheel, and the wheel can function without it. This unit will be defined as an "XZ liquid handler" and discussed in the next section.
Thus, when we define an object belonging to the wheel class, all the above information for the motors and the analogue ports should be clarified. One example to define an object of the wheel class is shown below:

```
WheelRight: # custom name
  class: Wheel
  devices:
    wheel_motor: # predefined name in Wheel
      board: board_2
      command_id: X_2
      config:
        reverted_switch: true
        reverted_direction: true
        enabled_acceleration: false
        speed: 5000
        max_speed: 12000
        homing_speed: 5000
        acceleration: 5000
    stirrer_motor: # predefined name in Wheel
      board: board_2
      config:
      command_id: A2_2
```

In the example, we defined an object called "WheelRight" from the class "Wheel". We need to define the following devices:

1. wheel_motor: the one that controls the Nema motor and rotates the wheel.
2. stirrer_motor: a write-analog port that turns on and off the stirring fans.

Again, we need to clarify the port and board for each device. It should be noted in this example, the driver types of both board_0 and board_2 should be Commanduino. The corresponding

"command_id" were used to clarify which ports from the boards were used. WheelLeft objects can be defined similarly.

1.2.2. XZ liquid handler

The design principle of the XZ liquid handler is to use a linear rail, lead screw, and a Nema motor to control the linear motion of the unit. For an XZ liquid handler, two motors are used to control the X and Z motion, respectively. There are two such designs as shown below:

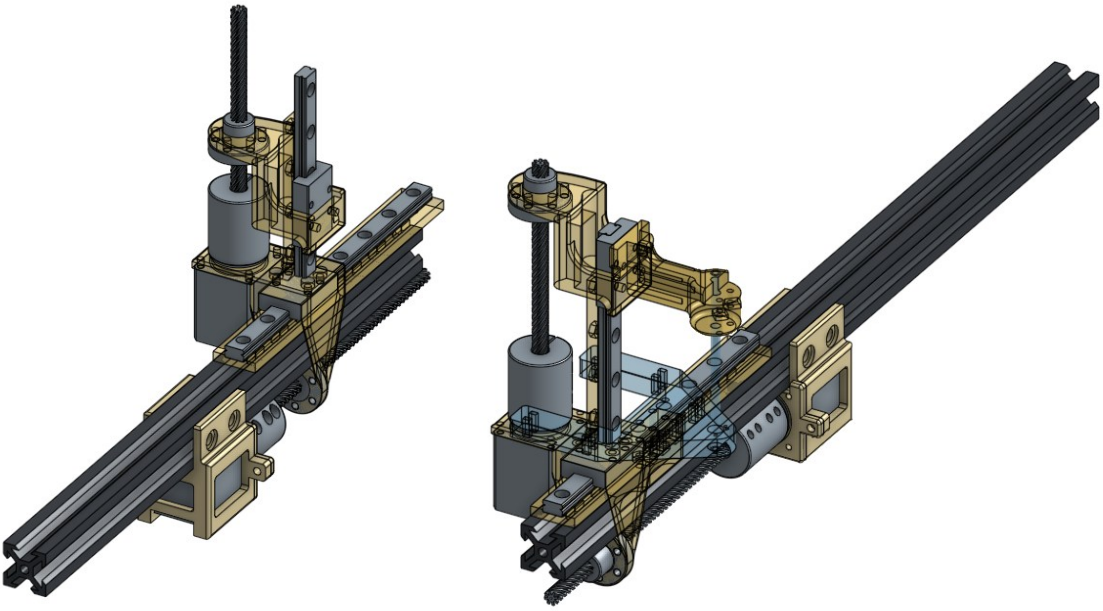

Figure S4: The CAD for the XZ liquid handler module.

The first one is to deal with the liquids of the 14 mL vials (e.g. on the wheel) and the right one is to manipulate the liquid of the UV-Vis cell. The size of the UV-Vis cell is small, so we need to rely on a small glass tube to reach the liquid.

The necessary drivers were defined as following:

1. X_motor, which is controlled by a Commanduino board.
2. Z_motor, which is controlled by a Commanduino board.
3. tricont_pumps, which is a series of pumps; each pump is controlled by a tricont board.

In the system, several XZ liquid handlers are defined. Here we simply list their names on this document:

1. XZLiquidHandlerLeft: The XZ liquid handler used together with the left wheel.

2. XZLiquidHandlerRight: The XZ liquid handler used together with the right wheel.
3. XZLiquidHandlerUVViS1/ XZLiquidHandlerUVViS2: the XZ liquid handlers used within the UV-Vis module.

1.2.3. Dispensing Unit

By now, we have introduced the wheel and the XZ liquid handler modules. We aim to mix the solutions using the vials in the wheel to conduct synthesis, but purely relying on the XZ liquid handler to mix the solutions is not practical – there are only limited tube channels in the XZ liquid handler, which constrains the number of reagents we can use. Thus, the dispensing unit was introduced for liquid mixing, which is shown below.

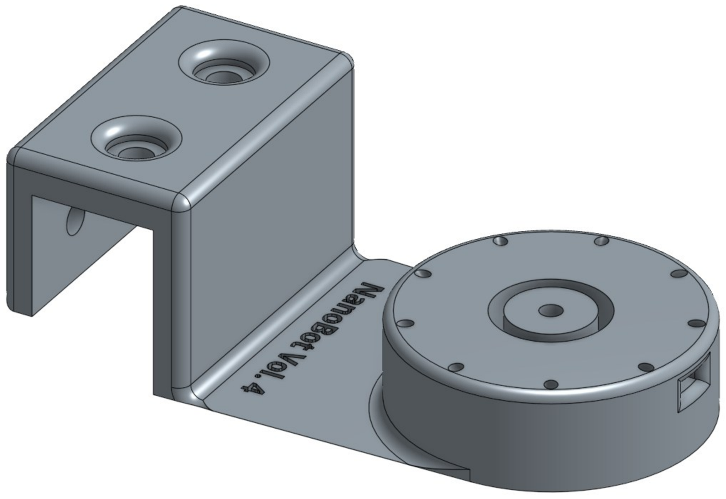

Figure S5: The CAD for the liquid dispensing module.

Many tubes can be inserted into the channels of this unit. Each of the tubes is connected to a tricont pump, while the tricont pump is connected to stock solutions. Thus, tricont pumps can transfer the stock solutions to the vials through this dispensing unit.

The definition of the dispensing unit is relatively simple: it is only related to a series of pumps, so we just need to define these pumps. An example is given below:

```yaml
DispensingUnitLeft:
  class: DispensingUnit
  devices:
    tricont_pumps: # predefined name
      pump_CTAB:
        switch: 0
        board: board_3
        top_velocity: 15000
        volume: 5.0
        initialize_valve_position: I
      pump_AA:
        switch: 1
        board: board_3
        top_velocity: 15000
        volume: 2.5
        initialize_valve_position: I
      pump_Au:
        switch: 4
        board: board_3
        top_velocity: 15000
        volume: 2.5
        initialize_valve_position: I
      pump_Ag:
        switch: 5
        board: board_3
        top_velocity: 15000
        volume: 2.5
        initialize_valve_position: I
      pump_KI:
        switch: 6
        board: board_3
        top_velocity: 15000
        volume: 1.0
        initialize_valve_position: I
      pump_Pt:
        switch: 7
        board: board_3
        top_velocity: 15000
        volume: 2.5
        initialize_valve_position: I
      pump_water:
        switch: 9
        board: board_3
        top_velocity: 15000
        volume: 5.0
        initialize_valve_position: I
```

We defined an object called “DispensingUnitLeft”, from the class of “DispensingUnit”. We need to define a series of pumps in “tricont_pumps” as the devices. As an example, we defined 7 pumps (pump_CTAB, pump_AA, pump_Au, pump_Ag, pump_KI, pump_Pt, and pump_water), which means they have a tube connected to the dispensing unit.

1.2.4. Hotplate matrix

We used a custom-built metal block as the heating mantle to heat the solutions in batches (shown below):

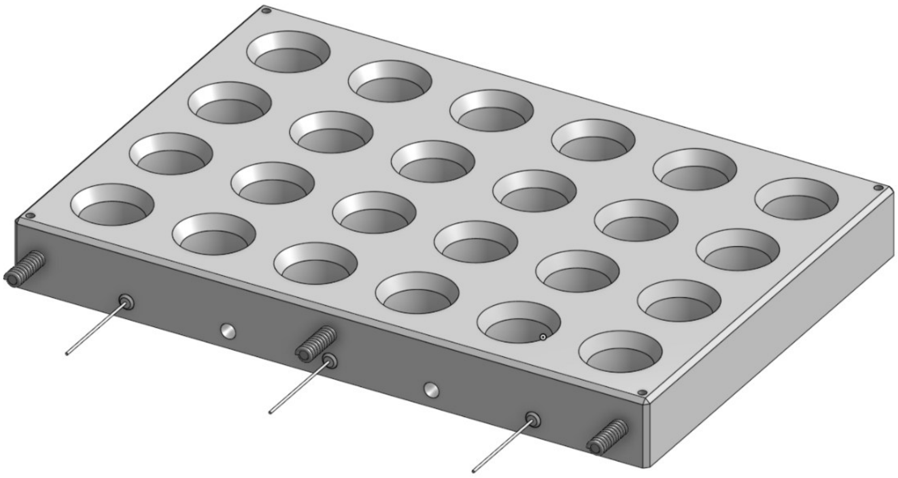

Figure S6: The CAD for the heating block.

There are 24 vial slots in the heating mantle. The temperature of the heating mantle is controlled by an Arduino board, which turns on the heating if the temperature is lower than the target temperature and turns off if the temperature is higher than the target one. Thus, we need two types of components to control the temperature.

The first type of component is the resistor, and the second is a temperature sensor. When powered, the current passes through the resistor generating heat (IR^2). Thus, we simply connected multiple resistors, placed in the holes of the metallic mantle to heat it. The on/off of the heating mantle is controlled by a MOSFET connected through an analogue port on the Arduino board. (https://kunkune.co.uk/shop/relay/dual-mosfet-driver-3-3-20v-trigger-15a-400w-out/?gclid=Cj0KCQiAutyfBhCMARIsAMgcRJQfrivAQ_nVzMA-lGf36V2Kbb4mYQK1MwAseDSme73nIrdJZkTKa60aAuKsEALw_wcB).

To control the temperature, we place three K-type thermal couplers into the heating mantle at the middle and both ends. The combination of K-type thermal couplers, MCP-9600 chips, and a normal Arduino board can be used to read out the temperature (see instructions here: https://learn.adafruit.com/adafruit-mcp9600-i2c-thermocouple-amplifier/arduino).

This script offers the codes to read the temperature of a single thermal coupler only. However, we can write our codes to:

1. Read the temperature of multiple tensors and average them.

2. Decide if we should turn on the MOSFET for heating or not according to the current average temperature and target temperature.

All these controlling logics are handled within the Arduino board in the current design; alternatively, we can also read the temperature, make a decision from Python, and send commands from Python to Arduino – however, consistently reading/writing from Arduino to Python and from Python to Arduino can be unstable.

Additionally, below the 24-vial slots, we also place the fans and the magnets, which can be used to stir the solutions during the heating process.

Thus, the configuration of this module is shown below:

```
HotPlate: #custom name
  class: HotPlateMatrix
  devices:
    stirerr_motor: # predefined name
      command_id: A2_5
      board: board_5
      config:
    temperature_controller:
      board: board_6
```

1.2.5. Capping unit

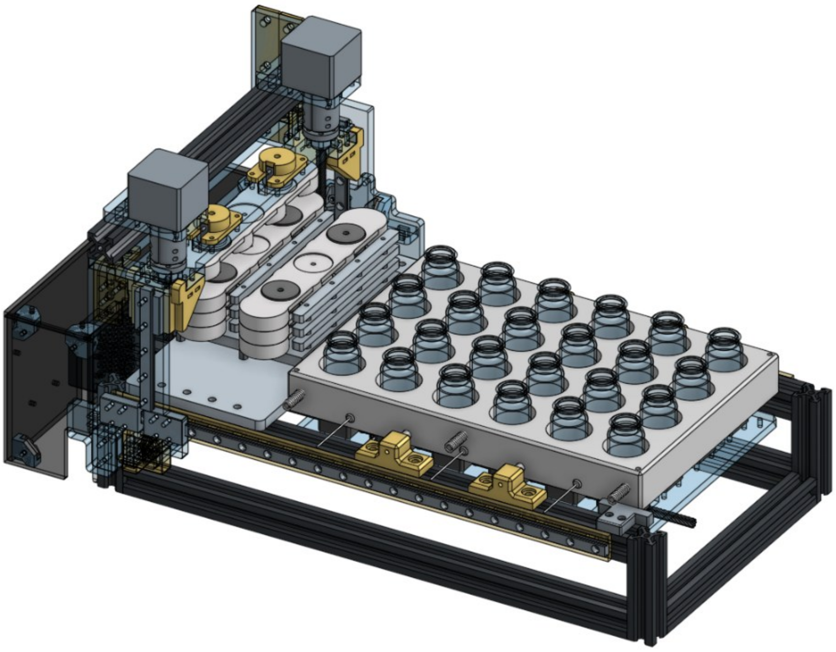

Figure S7: The CAD for the capping module with the heating block and stirring fans below.

Together with the heating mantle, we used an XZ capping unit to move the caps to the vial. The heating can cause the evaporation of solvents inside the vial, while putting a cap on top can avoid dramatic evaporation. All the caps are made of PTFE, which is very chemically resistant. We attached magnets on the top of the caps, which will be used to move the caps around.

There are three important components for the capping units:

1. An X_motor that controls the motion of the whole unit along the X-axis.
2. A Z_motor that controls the motion of the unit up and down.
3. A port that controls the on/off of the electric magnets. When they are turned on, the electric magnets can attach to the magnets on the top of the caps, so that we can capture the caps and move them around.

Thus, the configuration of the capping unit can be straightforward:

```
XZCappingModule:
  class: XZCappingModule
  devices:
    X_motor: # predefined name
      command_id: X_5
      board: board_5
      config:
        reverted_switch: true
        reverted_direction: false
        enabled_acceleration: false
        speed: 12000
        max_speed: 12000
        homing_speed: 8000
        acceleration: 8000
    Z_motor: # predefined name
      command_id: E0_5
      board: board_5
      config:
        reverted_switch: true
        reverted_direction: false
        enabled_acceleration: false
        speed: 8000
        max_speed: 8000
        homing_speed: 8000
        acceleration: 5000
    magnet:
      command_id: A1_5
      board: board_5
      config:
```

We only need to define the X/Z motors to control the movement of the unit, and an additional analogue port to turn on/off the magnets. The types of all the boards involved in the definition are "Commanduino".

1.2.6. UV-Vis module

There are two parts of the UV-Vis module: the laser and the spectrometer to detect the signals. The spectrometer is from Ocean Optics, and there is an online Python package to control the spectrometer directly. The Seabreeze will automatically detect the spectrometer once connected. (Seabreeze, https://python-seabreeze.readthedocs.io/en/latest/).

Controlling the laser is relatively simple, as the activation of the laser is controlled by a button. However, this doesn't allow us automated control of the system. Therefore, we modified the circuit by inserting a relay module that can be powered by an Arduino port. When powered, it closes the circuit, mimicking the situation when the button is switched off. By doing so, we can control the state of the laser through Arduino. Because two buttons are used to control the states for the UV light source and the Visible light source, we placed two relays to control the states of the whole system.

The configuration of the UV-Vis module is shown below:

```
QEPro2192UVVisModule:
  class: QEPro2192UVVisModule
  devices:
    QEPro2192:
      integration_time: 0.015
      board: board_11
    uv_source: # predefined name
      command_id: A1
      board: board_8
      config:
    vis_source: # predefined name
      command_id: A2
      board: board_8
      config:
```

The spectrometer is clarified by "board_11", type "QEPro2192". We used "board_8" to control the on/off for the UV and Vis light sources with a board type of Commanduino. It should be noted, this Arduino board does not require a RAMP because we only use it to write analogue signals.

1.2.7. XYZ gripping arm

The gripping arm is used to transfer vials from one module to another. It consists of four motors in total: three motors for the X, Y, and Z motion of the gripping unit, and one motor to control the gripping or releasing of the vials. Thus, its configuration can be simply set as below:

```yaml
XYZGripper:
  class: XYZGripper
  devices:
    X_motor: # predefined name
      command_id: X_0
      board: board_0
      config:
        reverted_switch: true
        reverted_direction: true
        enabled_acceleration: false
        speed: 4000
        max_speed: 4000
        homing_speed: 4000
        acceleration: 5000
    Y_motor: # predefined name
      command_id: E1_0
      board: board_0
      config:
        reverted_switch: true
        reverted_direction: false
        enabled_acceleration: false
        speed: 4000
        max_speed: 4000
        homing_speed: 4000
        acceleration: 5000
    Z_motor: # predefined name
      command_id: Z_0
      board: board_0
      config:
        reverted_switch: true
        reverted_direction: true
        enabled_acceleration: false
        speed: 12000
        max_speed: 12000
        homing_speed: 6000
        acceleration: 5000
    G_motor:
      command_id: E0_0
      board: board_0
      config:
        reverted_switch: true
        reverted_direction: true
        enabled_acceleration: false
        speed: 6000
        max_speed: 6000
        homing_speed: 4000
        acceleration: 2000
```

1.3. XDL steps and basic steps

The following is an introduction to the steps with clarified logic, but the actual implementation of the logic should be found in the source code.

1.3.1. Add

The Add step is used to add a certain amount of reagent to a vessel. It relies on a basic step of "MWAdd". The logic for the basic step is as follows:

1. Check if we are adding a solution through "DispensingUnit" or "LiquidHanlder".
2. Check if we need to stir the solution or not. If so, set the stirring speed.
3. Check the current position of the vessel. If it is on a wheel, the wheel should be rotated so that the vessel is below the dispensing unit or can be accessed by the liquid handler.
4. If the addition will be achieved by the dispensing unit, find the pump that connects the dispensing unit and the reagent. Add the solution through the pump at a specified speed.
5. If the addition will be achieved by the liquid handler, move the X/Y/Z motor of the liquid handler to access the vessel. Then find the pump that connects the liquid handler and the reagent, and add the solution through the pump at a specified speed.

1.3.2. Transfer

The Transfer step is used to transfer a solution from one vessel to another vessel. It relies on a basic step of "MWTransfer". The basic logic is as follows:

1. Check if we will use a pair of liquid handlers or just a single liquid handler.
2. Check the positions of the from_vessel and the to_vessel that will be involved in the liquid transfer process.
3. Move the liquid handler to access both vessels according to the positions.
4. Find the pump that connects the liquid handlers and vessels and transfer the solution at a specific speed.

1.3.3. TransferVessel

The TransferVessel step is used to transfer a vessel from one position to another. The vessels can be transferred as a batch or one by one. Since we will cap a batch of four vessels on the heating mantle, we usually transfer the vessels with a batch number of 4. The "TransferVessel" step relies on a basic step of "MWTransferVial". The basic logic is as follows:

1. Check if we have any gripping arms in the system and get the available gripping arm.
2. Check the current position of the vial and see if the gripping arm can reach it.
3. Check the target position and if the gripping arm can reach the target position.
4. Pick up the vessel using the gripping arm.
5. Release the vessel to the target position.

1.3.4. SealVessel

After transferring the vessels to a heating mantle, we need to seal the vessels to avoid evaporation of the solutions. The SealVessel step was used for this purpose, and the basic step is MWCapVial. Its basic logic is as follows:

1. Check the position of the current vessel. If it is on a heating mantle with a capping mechanism, we will cap the vessel.
2. Cap the vessel according to the position of the vessel.

1.3.5. UnsealVessel

Similar to the SealVessel step, the basic logic of "UnsealVessel" with a basic step of MWUncapVial is as follows:

1. Check the position of the current vessel. If it is on a heating mantle with a capping mechanism, we will cap the vessel.
2. Uncap the vessel according to the position of the vessel.

1.3.6. HeatChillToTemp

We have a heating mantle to heat the solution of the vessels. This step was used to control its temperature. The corresponding basic step is "MWHeatChillTo". Its basic logic is as follows:

1. Check the position of the reactor to see if it is on a heating mantle module.
2. If the reactor is on the heating mantle module, set the temperature of the heating mantle module. Otherwise, tell the user the reactor cannot be heated up.

1.3.7. StartStir

StartStir is used to turn on the stirring fans below the vessels. It uses the basic step of "MWSetStir", whose logic is as below:

1. Find the location of the vessels.
2. Check if there are any stirring fans in the module where the vessel is located. If so, turn on the stirring fans to the specified speed.

1.3.8. StopStir

StartStir is used to turn off the stirring fans below the vessels. It uses the basic step of “MWSetStir”, with the stirring speed specified as “0”.

1.3.9. TakeUVVis

This step was used for the UV-Vis characterization of the samples. There are three modes: “reference”, “single sample”, and “multiple samples” respectively. When reference mode was used, the spectrometer will record the raw signal of the spectrum, while for the rest two modes, the raw signal and the absorption intensities will be recorded. When we calculate the absorption intensity, a reference spectrum is required, and by default, we use the latest reference spectrum. In “multiple sample” mode, the spectra will be taken every few seconds (which can be specified) and these multiple spectra will be recorded. The basic step for “TakeUVVis” is MWRecordUVVis.

2. Design an experiment using XDL

We write a series of XDL steps in the .xdl file and execute these steps. When the XDL steps were executed, they essentially utilized a lower layer called "XDL basic steps". The XDL basic steps communicate with the Python interface to control the hardware and perform the experiments. The relation between XDL steps, XDL basic steps, Python API (discussed above), and the hardware is shown in the figure below.

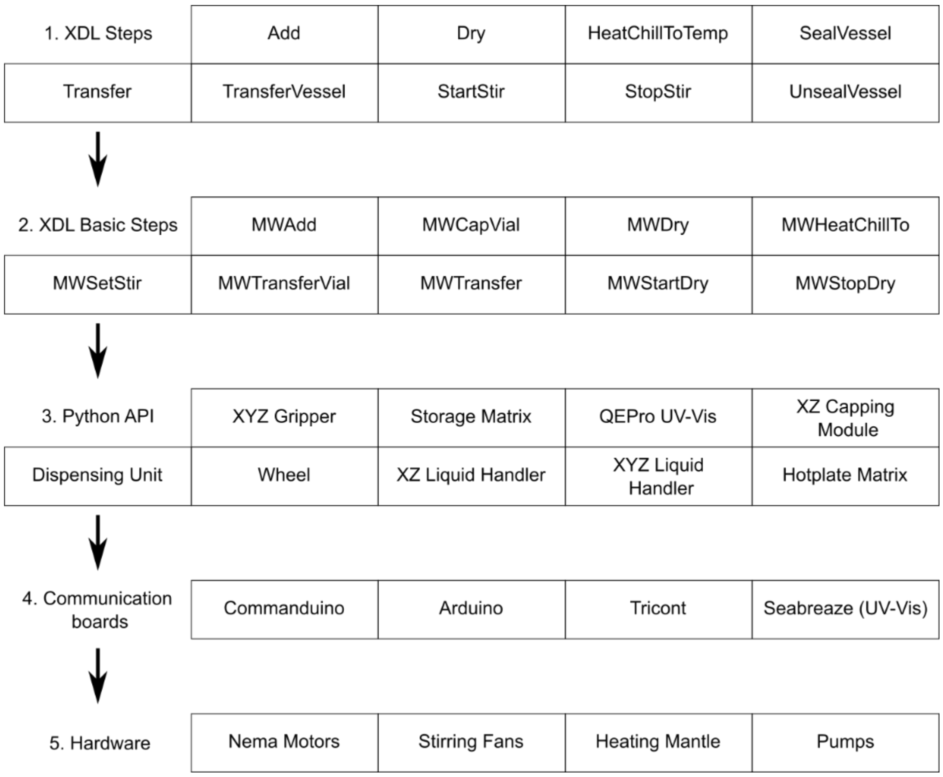

Scheme S1: The software control logic of the modular platform.

2.1. Nanoparticles Synthesis

Each experiment was programmed using an XDL script divided into three main parts **(Figure S9)**: (a) Definition of all reactors, components, and reagents involved in the synthesis. (b) Execution of the synthesis procedure, including the precise addition of each reagent into designated vials positioned on the Geneva wheel. (c) Process control operations, such as stirring, transferring vials to the heating matrix, maintaining the reaction at elevated temperature for 64,800 seconds (18 hours), and returning the vials to the Geneva wheel.

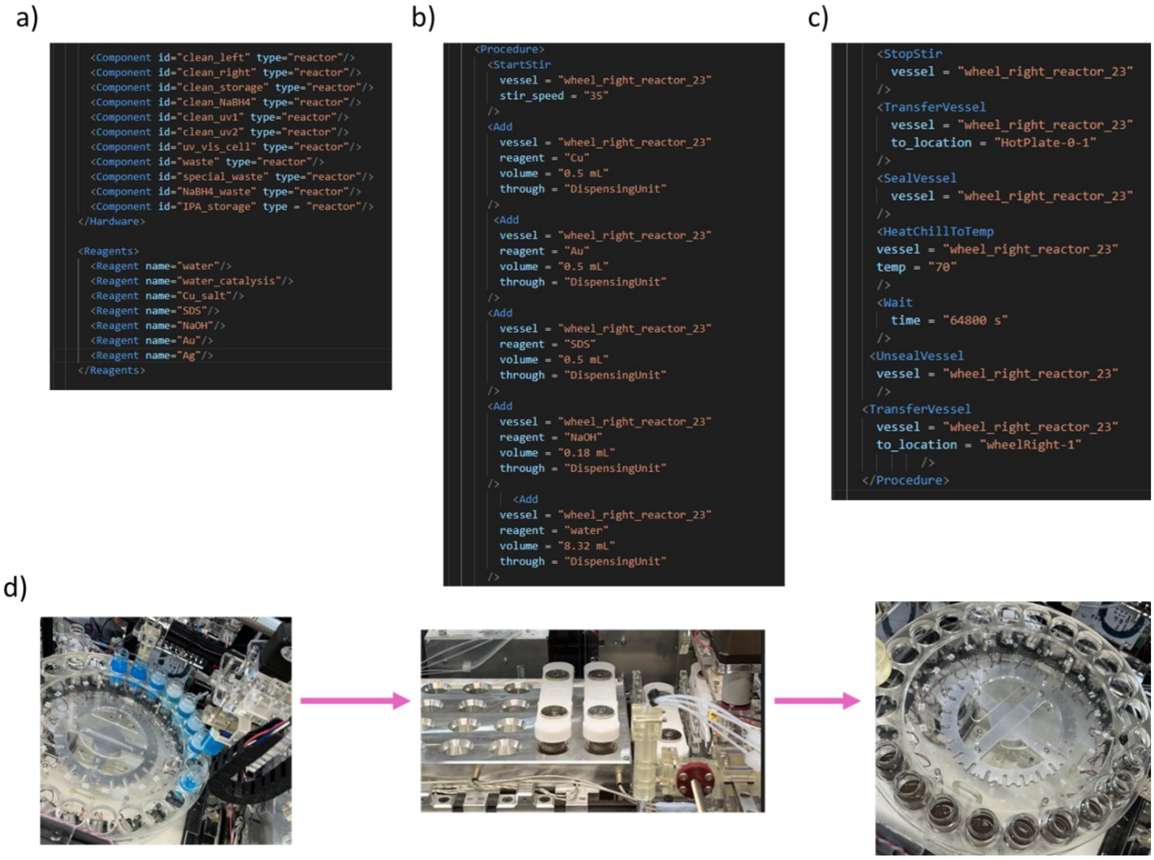

Figure S9: Synthesis methods mediated by XDL. a) Components and reagents identification. **b)** Starting material to be added from the stock solution to a vial. **c)** Synthesis parameters including stirring, heating, and transferring. **d)** Example of CuO-based NP synthesis, showing processing via Geneva wheels and hotplate matrix.

2.2. Photocatalytic Activity assay

For the exploration of the photocatalytic activity of Methyl Green, we implemented an in-line UV-Vis spectroscopy station and conducted the following XDL steps for each measurement as follows in **Figure S10**: (a) Turn on the UV-Vis bulb and record a spectrum in "reference mode" to form a baseline. Two operational modes were used: "Reference mode": Captures a raw spectrum as a baseline. "Multiple samples mode": Collects spectra at defined intervals and converts them into real-time absorption intensity graphs. (b) Clean the UV-Vis cell three times before measurement. (c) Take another UV-Vis reference mode measurement after cleaning for validation. (d) Transfer the sample from a specific vial on the Geneva wheel (e.g., position 23) to the UV-Vis cell for measurement in "multiple samples mode". (e) Git rid of the measured sample to waste and clean the UV-Vis cell three times with water to prepare the cell for the next measurement. Finally, PNG and JSON files were collected for further analysis.

a)

```
<!--Turn on UV-Vis now and get everything ready-->
<TakeUVVis
uv_source = "False"
vis_source = "True"
mode = "reference"
queue = "B"
/>
```

b)

```
<Add
vessel = "clean_right"
reagent = "water"
volume = "12 mL"
through = "LiquidHandler"
queue = "C3"
/>
<Transfer
from_vessel = "clean_right"
to_vessel = "uv_vis_cell"
volume = "2.7 mL"
through = "LiquidHandlerPair"
to_depth = "1"
/>
```

c)

```
<!--Take a UV-Vis reference after the cleaning process-->
<TakeUVVis
uv_source = "False"
vis_source = "True"
mode = "reference"
/>
```

d)

```
<Transfer
from_vessel = "wheel_right_reactor_23"
to_vessel = "uv_vis_cell"
volume = "1.5 mL"
from_depth = "0.70"
to_depth = "0.0"
through = "LiquidHandlerPair"
/>
<Transfer
from_vessel = "uv_vis_cell"
to_vessel = "waste"
volume = "2 mL"
through = "SingleLiquidHandler"
/>
<Transfer
from_vessel = "wheel_right_reactor_23"
to_vessel = "uv_vis_cell"
volume = "2.5 mL"
from_depth = "0.70"
to_depth = "0.0"
through = "LiquidHandlerPair"
/>
<TakeUVVis
uv_source = "False"
vis_source = "True"
mode = "multiple samples"
/>
```

e)

```
<Repeat repeats = "3" queue = "B">
  <!--Clean the uv_vis line-->
  <Transfer
  from_vessel = "uv_vis_cell"
  to_vessel = "waste"
  volume = "3 mL"
  through = "SingleLiquidHandler"
  queue = "C1"
  />
```

Figure S10: XDL steps for operating the in-line UV-Vis spectroscopy station.
(a-e) Including reference measurement, sample transfer, sample measurement, and cleaning procedures

3. Characterization

3.1. Validation: CuO-based NPs synthesis

Synthesis validation was conducted using four main parameters: appearance, crystal structure, morphology, and homogeneity (based on DLS measurements), to compare CuO-based NPs produced by the robotic platform and manually:

CuO NPs		
**Parameters**	**Robotic platform**	**Manual work**
Appearance	Dark brown	Dark brown
Crystal structure	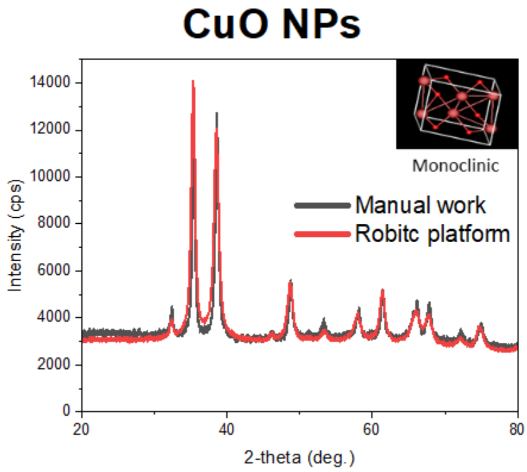	
Morphology	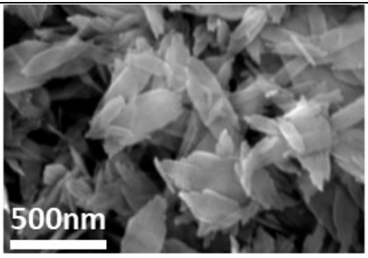	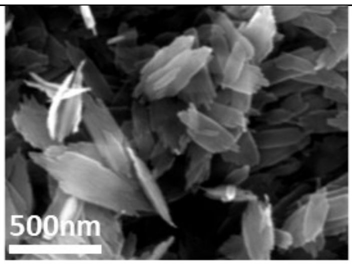
Homogeneity	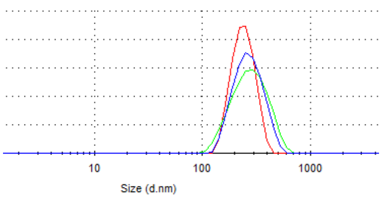	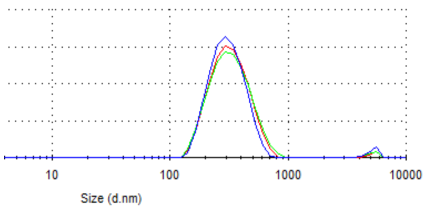

Table S1: Comparative characterization of CuO nano-leaves synthesized manually versus by the robotic platform, including sample appearance, XRD patterns, SEM morphology, and homogeneity.

CuO-Au NPs		
**Parameters**	**Robotic platform**	**Manual work**
Appearance	Dark brown	Dark brown
Crystal structure	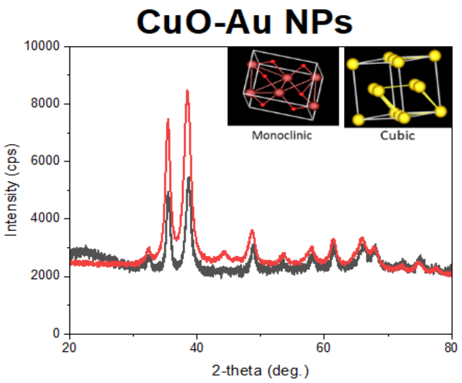	
Morphology	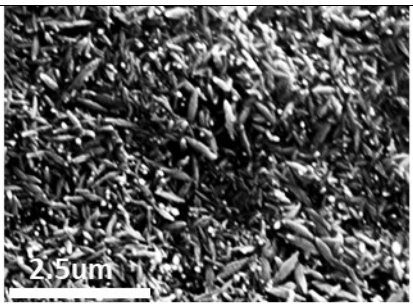	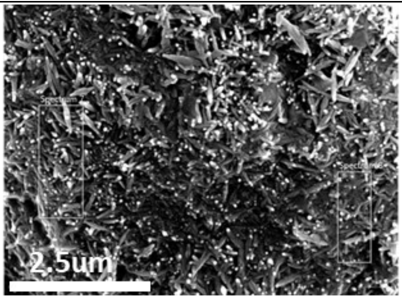
Homogeneity	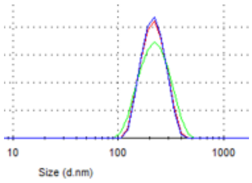	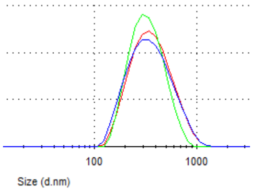

Table S2: Comparative characterization of CuO-Au toothpick-like shape NPs synthesized manually versus by the robotic platform, including sample appearance, XRD patterns, SEM morphology, and homogeneity.

<table>
<tr><th colspan="3">$CuO-Ag_2O$ NPs</th></tr>
<tr><th>Parameters</th><th>Robotic platform</th><th>Manual work</th></tr>
<tr><td>Appearance</td><td>Dark brown</td><td>Dark brown</td></tr>
<tr><td>Crystal structure</td><td colspan="2">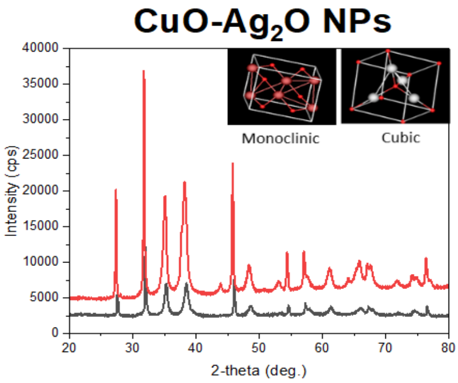
</td></tr>
<tr><td>Morphology</td><td>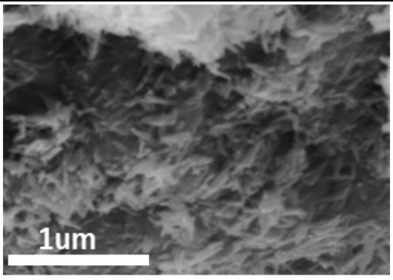
</td><td>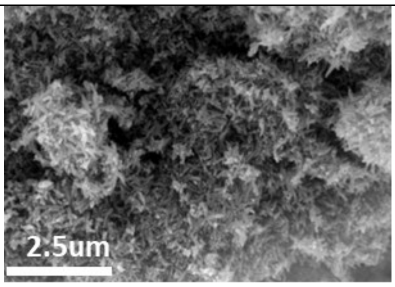
</td></tr>
<tr><td>Homogeneity</td><td>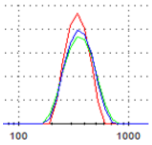
</td><td>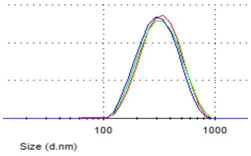
</td></tr>
</table>

Table S3: Comparative characterization of $CuO-Ag_2O$ toothpick-like shape NPs synthesized manually versus by the robotic platform, including sample appearance, XRD patterns, SEM morphology, and homogeneity.

3.2. Trials: TiO_2-based NPs synthesis

Paramaters	TiO_2 synthesis	TiO_2-Au synthesis
XRD pattern	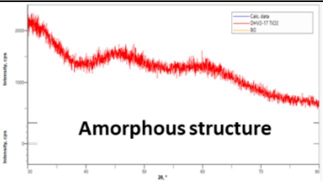 No diffraction pattern	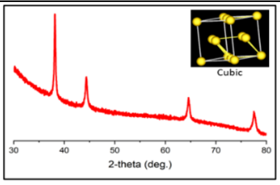 AuNPs XRD pattern
Appearance	White powder	Pink suspension with black precipitation
Morphology	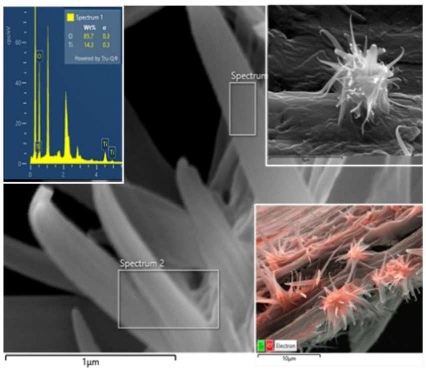 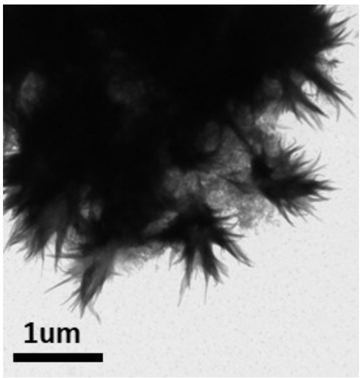	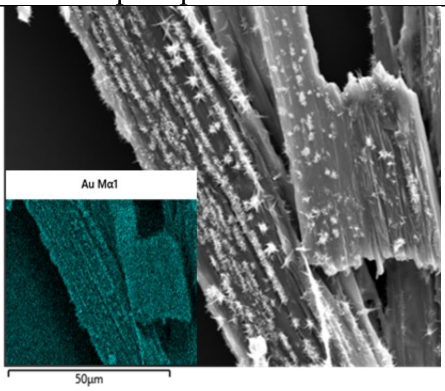 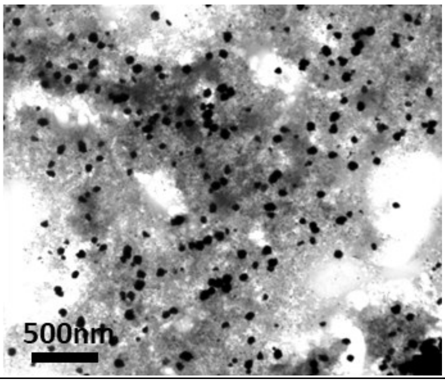

Table S4: Trials of TiO2-based NP synthesis using the robotic platform. Sample appearance, XRD patterns, and SEM morphology indicate that the one-pot method successfully applied for CuO-based NPs is not suitable for Ti, producing an amorphous material. However, in the presence of Au precursor, crystalline AuNPs were deposited on the amorphous TiO_2.

3.3. Trials: NiO-based NPs synthesis

Parameters	NiO synthesis	CuO-NiO synthesis
Appearance	White powder	Brown powder
Morphology	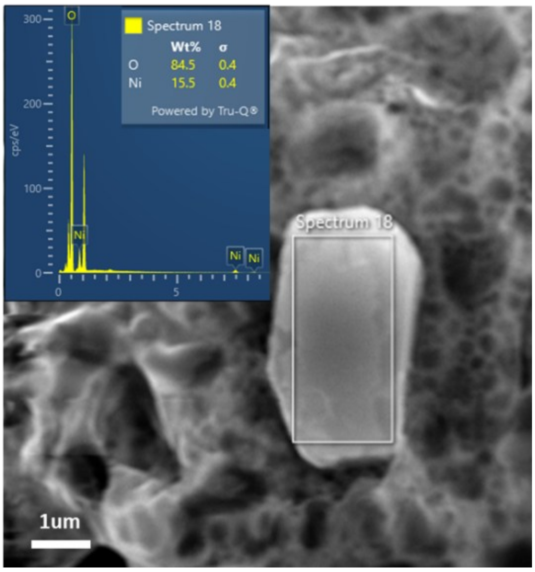	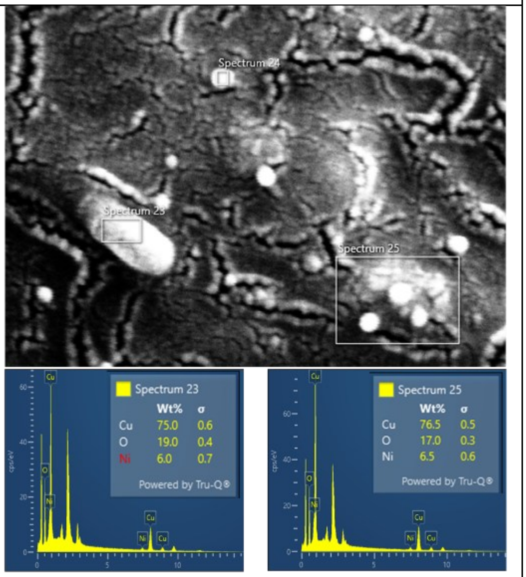

Table S5: Trials of NiO-based NP synthesis using the robotic platform. Sample appearance and SEM morphologies were enough to indicate that the one-pot method successfully applied for CuO-based NPs is not suitable for Ni, producing an amorphous material.

3.4. Validation: in-line UV-Vis spectroscopy station

The in-line UV-Vis spectroscopy was validated against the plate reader using plasmonic Au-Ag nanoparticles. Eight reactions were carried out on the robotic platform, varying AgNP seeds with added $HAuCl_4$ precursor, with a constant amount of ascorbic acid.[1,2] The absorbance spectrum of each sample was measured once after synthesis using the in-line UV-Vis and then with the plate reader. This comparison confirmed that absorbance measurements were consistent and reliable across both instruments, due to the distinct plasmonic peaks of the metals, with the results shown below:

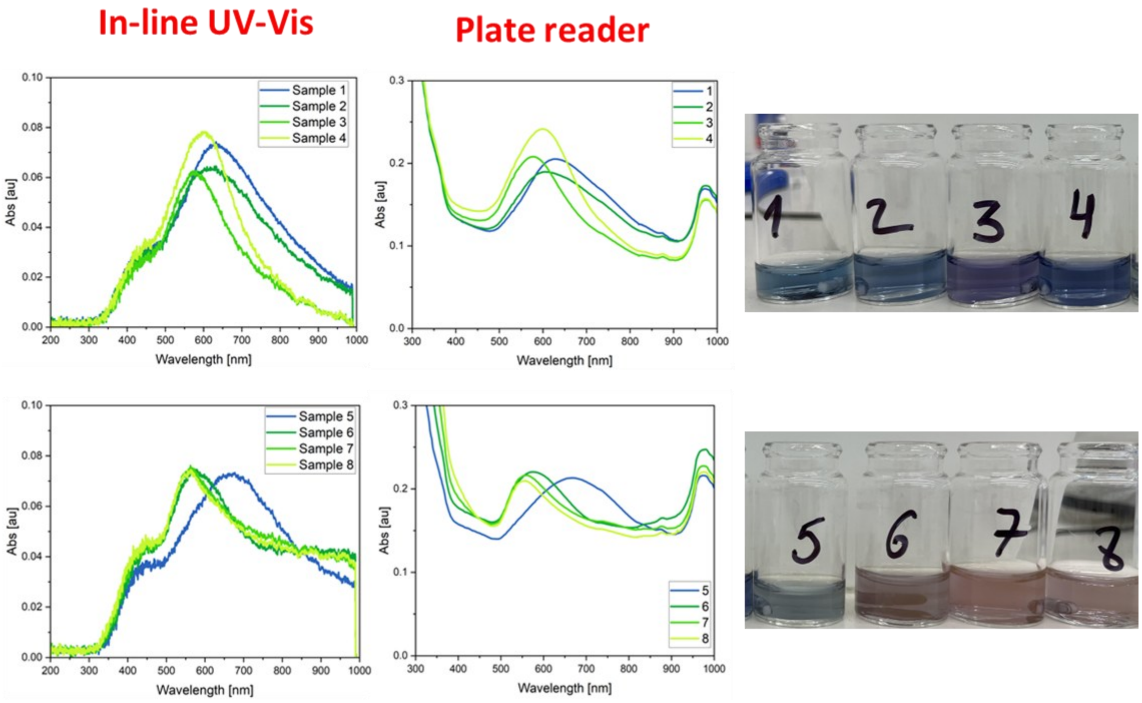

Figure S11: Comparative absorbance spectra of Ag-Au NPs. Samples were synthesized by the robotic platform, and absorbance was measured using both the in-line UV-Vis and the plate reader, demonstrating consistent and reliable data.

3.5. Powder X-ray diffraction – additional data

CuO NPs

No.	2θ, °	d, Å	Height, cps	FWHM, °	Int. I., cps°	Int. W., °	Asymmetry	Decay(ηL/mL)	Decay(ηH/mH)	Size, Å
2	6.710(12)	13.16(2)	1201(16)	0.48(2)	1235(25)	1.03(3)	0.79(10)	1.55(8)	1.55(7)	172(8)
3	32.258(6)	2.7728(5)	396(7)	0.456(17)	211(9)	0.53(3)	1.05(19)	0.49(14)	0.0(2)	189(7)
4	35.280(3)	2.5419(2)	7084(70)	0.614(3)	5907(17)	0.834(11)	1.15(2)	0.641(14)	0.707(16)	141.9(6)
5	38.455(3)	2.3390(2)	5652(60)	0.776(3)	6049(16)	1.070(14)	0.976(19)	0.716(14)	0.699(13)	113.2(5)
6	46.08(8)	1.968(3)	134(3)	0.62(9)	114(12)	0.85(10)	1.3(8)	1.0(4)	0.0(7)	145(21)
7	48.597(7)	1.8720(3)	1543(24)	0.853(7)	1720(11)	1.11(2)	0.93(3)	0.82(3)	0.29(3)	106.7(8)
8	53.33(4)	1.7163(11)	211(4)	0.78(3)	175(9)	0.83(6)	1.0(2)	0.0(3)	0.0(3)	119(5)
9	57.903(15)	1.5913(4)	706(13)	1.021(15)	1049(10)	1.49(4)	1.10(8)	0.91(5)	0.75(5)	92.8(14)
10	61.252(8)	1.51207(18)	1342(22)	0.798(8)	1360(10)	1.01(2)	0.65(3)	1.01(4)	0.00(4)	120.8(12)
11	65.974(11)	1.4148(2)	782(14)	1.31(2)	1112(17)	1.42(5)	1.24(7)	0.00(4)	0.13(4)	75.4(11)
12	67.723(10)	1.38248(17)	710(13)	1.13(2)	871(16)	1.23(4)	1.24(7)	0.00(4)	0.13(4)	88.4(18)
13	72.156(9)	1.30806(14)	201(4)	0.883(17)	255(4)	1.27(4)	1.62(6)	0.00(8)	1.48(4)	116(2)
14	74.615(4)	1.27093(6)	584(11)	1.063(12)	729(8)	1.25(4)	0.51(4)	0.73(4)	0.00(5)	98.2(11)
15	80.04(3)	1.1979(3)	90(2)	1.28(7)	123(6)	1.37(9)	0.83(8)	0.00(18)	0.00(10)	84(4)
16	82.568(12)	1.16749(14)	226(5)	1.82(4)	438(7)	1.94(7)	0.83(8)	0.00(18)	0.00(10)	60.6(12)
17	89.78(4)	1.0914(4)	68.6(16)	1.10(8)	103(5)	1.50(11)	1.8(3)	0.0(3)	1.38(16)	107(8)
18	98.54(10)	1.0165(8)	101(2)	2.21(10)	254(11)	2.51(17)	0.9(2)	0.4(2)	0(2)	58(3)

No.	2θ, °	Phase Name	Chemical Formula	Card No	Norm. I.	Profile Type	Distributi...
2	6.710(12)	Unknown			20.42	Split pseudo-Voigt	-
3	32.258(6)	Tenorite: 1 1 0	Cu O	1011194	3.49	Split pseudo-Voigt	-
4	35.280(3)	Tenorite: 0 0 2	Cu O	1011194	97.66	Split pseudo-Voigt	-
5	38.455(3)	Tenorite: 1 1 1	Cu O	1011194	100.00	Split pseudo-Voigt	-
6	46.08(8)	Tenorite: 1 1 -2	Cu O	1011194	1.88	Split pseudo-Voigt	-
7	48.597(7)	Tenorite: 2 0 -2	Cu O	1011194	28.43	Split pseudo-Voigt	-
8	53.33(4)	Tenorite: 0 2 0	Cu O	1011194	2.90	Split pseudo-Voigt	-
9	57.903(15)	Tenorite: 2 0 2	Cu O	1011194	17.35	Split pseudo-Voigt	-
10	61.252(8)	Tenorite: 1 1 -3	Cu O	1011194	22.48	Split pseudo-Voigt	-
11	65.974(11)	Tenorite: 3 1 -1	Cu O	1011194	18.39	Split pseudo-Voigt	-
12	67.723(10)	Tenorite: 1 1 3	Cu O	1011194	14.40	Split pseudo-Voigt	-
13	72.156(9)	Tenorite: 3 1 1	Cu O	1011194	4.22	Split pseudo-Voigt	-
14	74.615(4)	Tenorite: 0 0 4	Cu O	1011194	12.05	Split pseudo-Voigt	-
15	80.04(3)	Tenorite: 1 1 -4	Cu O	1011194	2.04	Split pseudo-Voigt	-
16	82.568(12)	Tenorite: 2 2 2	Cu O	1011194	7.25	Split pseudo-Voigt	-
17	89.78(4)	Tenorite: 1 3 -1	Cu O	1011194	1.70	Split pseudo-Voigt	-
18	98.54(10)	Tenorite: 0 2 4	Cu O	1011194	4.20	Split pseudo-Voigt	-

Lattice parameters

Phase name	a, Å	b, Å	c, Å	α, °	β, °	γ, °
Tenorite	4.69296	3.44686	5.14517	90.000	99.530	90.000

d-I List

Tenorite

No.	2θ, °	d, Å	h k l	Norm. I.
1	32.35873	2.76443	1 1 0	7.37
2	35.34977	2.53708	0 0 2	27.70
3	35.37750	2.53515	1 1 -1	77.79
4	38.56607	2.33257	1 1 1	100.00
5	38.88634	2.31409	2 0 0	21.23
6	46.06718	1.96870	1 1 -2	2.09
7	48.62694	1.87088	2 0 -2	29.06
8	51.17943	1.78341	1 1 2	1.37
9	53.09703	1.72343	0 2 0	10.67
10	56.33263	1.63187	0 2 1	0.85
11	58.19046	1.58412	2 0 2	15.17
12	61.30863	1.51081	1 1 -3	20.45
13	65.41158	1.42562	0 2 2	16.41
14	66.07087	1.41298	3 1 -1	15.47
15	66.32806	1.40812	3 1 0	0.46
16	67.68521	1.38315	1 1 3	9.29
17	67.73725	1.38222	2 2 0	14.40
18	68.51677	1.36838	2 2 -1	0.37
19	71.48632	1.31866	3 1 -2	0.33
20	72.22920	1.30691	3 1 1	7.37
21	72.58456	1.30139	2 2 1	0.29
22	74.77908	1.26854	0 0 4	5.93
23	74.84562	1.26758	2 2 -2	6.33
24	79.30148	1.20716	0 2 3	0.21

No.	2θ, °	d, Å	h k l	Norm. I.
25	79.93321	1.19920	2 0 -4	2.01
26	79.96871	1.19876	1 1 -4	0.21
27	82.10990	1.17283	3 1 -3	4.77
28	82.67144	1.16629	2 2 2	4.55
29	83.29610	1.15912	3 1 2	0.18
30	83.47877	1.15705	4 0 0	4.06
31	86.30585	1.12625	2 2 -3	0.15
32	86.38404	1.12543	4 0 -2	1.59
33	87.38428	1.11511	1 3 0	0.00
34	87.68897	1.11202	1 1 4	0.16
35	89.06727	1.09834	1 3 -1	6.69
36	90.98478	1.08012	1 3 1	2.76
37	95.33204	1.04202	2 0 4	1.23
38	96.04227	1.03619	1 3 -2	0.00
39	97.76825	1.02245	3 1 -4	0.12
40	97.83740	1.02191	2 2 3	0.11
41	97.87411	1.02163	0 2 4	2.93
42	99.43424	1.00975	3 1 3	4.32
43	99.91535	1.00617	1 3 2	0.00

Table S6: Additional XRD data for CuO nano-leaf contains characteristic peaks corresponding to the databases and lattice parameters.

CuO-Au NP heterostructure

No.	2θ, °	d, Å	Height, cps	FWHM, °	Int. I., cps°	Int. W., °	Asymmetry	Decay(ηL/mL)	Decay(ηH/mH)	Size, Å
2	4.441(5)	19.88(2)	28564(143)	0.196(4)	6519(135)	0.228(6)	0.82(8)	0.53(10)	0.01(9)	424(9)
3	6.733(6)	13.117(12)	12293(89)	0.193(5)	2524(84)	0.205(8)	1.5(2)	0.00(13)	0.00(16)	431(12)
4	11.257(12)	7.854(8)	487(9)	0.72(6)	743(46)	1.53(12)	0.70(18)	1.55(9)	1.55(11)	116(10)
5	13.56(3)	6.525(14)	561(11)	0.88(6)	1046(38)	1.87(10)	1.1(2)	1.55(8)	1.55(9)	95(6)
6	32.38(3)	2.763(3)	301(6)	1.27(5)	760(13)	2.52(10)	2.1(3)	1.47(6)	1.35(12)	68(3)
7	35.325(3)	2.5388(2)	3176(43)	0.899(4)	3915(13)	1.23(2)	0.901(14)	0.890(12)	0.486(12)	96.9(5)
8	38.401(4)	2.3422(2)	3854(49)	1.225(5)	6475(16)	1.68(3)	0.901(14)	0.890(12)	0.486(12)	71.7(3)
9	43.56(9)	2.076(4)	45.6(10)	0.5(5)	37(65)	0.8(14)	0.6(4)	0.0(4)	1.55(15)	191(190)
10	44.24(9)	2.046(4)	137(3)	1.25(17)	297(61)	2.2(5)	0.6(4)	0.0(4)	1.55(15)	72(10)
11	48.632(13)	1.8707(5)	762(15)	1.224(13)	1277(10)	1.68(5)	1.37(7)	0.59(4)	0.82(4)	74.4(8)
12	53.489(11)	1.7117(3)	185(4)	1.02(3)	208(9)	1.13(7)	1.03(15)	0.20(16)	0.00(19)	91(3)
13	57.930(10)	1.5906(3)	258(5)	1.34(2)	372(5)	1.44(5)	1.37(3)	0.00(4)	0.10(5)	71.0(11)
14	61.420(6)	1.50834(14)	495(10)	1.038(10)	554(5)	1.12(3)	1.37(3)	0.00(4)	0.10(5)	93.0(9)
15	65.93(2)	1.4157(4)	567(11)	2.167(19)	1542(13)	2.72(8)	0.95(4)	0.30(4)	0.61(4)	45.6(4)
16	67.84(3)	1.3804(5)	316(7)	1.10(2)	371(10)	1.17(6)	0.72(7)	0.00(16)	0.00(12)	90.7(18)
17	72.06(4)	1.3096(6)	71.6(17)	1.02(8)	80(5)	1.12(10)	0.92(10)	0.0(2)	0.15(18)	100(8)
18	74.84(4)	1.2676(5)	187(4)	1.30(3)	266(8)	1.42(7)	0.92(10)	0.0(2)	0.15(18)	80(2)
19	77.45(3)	1.2313(5)	88(2)	0.97(6)	94(5)	1.06(8)	0.92(10)	0.0(2)	0.15(18)	109(7)
20	82.48(6)	1.1685(7)	138(3)	2.09(5)	306(11)	2.22(13)	0.96(11)	0.00(17)	0.00(17)	52.9(13)
21	89.97(5)	1.0896(5)	55.8(14)	2.43(9)	144(6)	2.58(17)	0.53(5)	0.0(3)	0.00(16)	48.3(19)
22	98.46(15)	1.0171(11)	46.9(12)	1.37(12)	72(7)	1.5(2)	0.9(4)	0.0(6)	0.2(15)	93(8)

No.	2θ, °	Phase Name	Chemical Formula	Card No	Norm. I.	Profile Type	Distributi...
2	4.441(5)	Unknown			100.00	Split pseudo-Voigt	-
3	6.733(6)	Unknown			38.72	Split pseudo-Voigt	-
4	11.257(12)	Unknown			11.39	Split pseudo-Voigt	-
5	13.56(3)	Unknown			16.05	Split pseudo-Voigt	-
6	32.38(3)	Tenorite: 1 1 0	Cu O	1011194	11.66	Split pseudo-Voigt	-
7	35.325(3)	Tenorite: 0 0 2	Cu O	1011194	60.06	Split pseudo-Voigt	-
8	38.401(4)	Gold: 1 1 1,Tenorite: 1 1 1	Au,Cu O	1100138,1011194	99.32	Split pseudo-Voigt	-
9	43.56(9)	Unknown			0.57	Split pseudo-Voigt	-
10	44.24(9)	Gold: 2 0 0	Au	1100138	4.55	Split pseudo-Voigt	-
11	48.632(13)	Tenorite: 2 0 -2	Cu O	1011194	19.59	Split pseudo-Voigt	-
12	53.489(11)	Tenorite: 0 2 0	Cu O	1011194	3.19	Split pseudo-Voigt	-
13	57.930(10)	Tenorite: 2 0 2	Cu O	1011194	5.71	Split pseudo-Voigt	-
14	61.420(6)	Tenorite: 1 1 -3	Cu O	1011194	8.50	Split pseudo-Voigt	-
15	65.93(2)	Gold: 2 2 0,Tenorite: 3 1 -1	Au,Cu O	1100138,1011194	23.65	Split pseudo-Voigt	-
16	67.84(3)	Tenorite: 2 2 0	Cu O	1011194	5.68	Split pseudo-Voigt	-
17	72.06(4)	Tenorite: 3 1 1	Cu O	1011194	1.23	Split pseudo-Voigt	-
18	74.84(4)	Tenorite: 2 2 -2	Cu O	1011194	4.08	Split pseudo-Voigt	-
19	77.45(3)	Gold: 3 1 1	Au	1100138	1.44	Split pseudo-Voigt	-
20	82.48(6)	Gold: 2 2 2,Tenorite: 2 2 2	Au,Cu O	1100138,1011194	4.70	Split pseudo-Voigt	-
21	89.97(5)	Tenorite: 1 3 1	Cu O	1011194	2.21	Split pseudo-Voigt	-
22	98.46(15)	Gold: 4 0 0,Tenorite: 0 2 4	Au,Cu O	1100138,1011194	1.10	Split pseudo-Voigt	-

Lattice parameters

Phase name	a, Å	b, Å	c, Å	α, °	β, °	γ, °
Gold	4.07110	4.07110	4.07110	90.000	90.000	90.000
Tenorite	4.69221	3.44632	5.14435	90.000	99.530	90.000

d-I List

Gold

No.	2θ, °	d, Å	h k l	Norm. I.
1	38.26122	2.35045	1 1 1	100.00
2	44.47189	2.03555	2 0 0	48.37
3	64.71074	1.43935	2 2 0	29.84
4	77.73790	1.22748	3 1 1	34.05
5	81.90686	1.17523	2 2 2	9.84
6	98.37337	1.01778	4 0 0	5.13

Tenorite

No.	2θ, °	d, Å	h k l	Norm. I.
1	32.36399	2.76400	1 1 0	7.37
2	35.35554	2.53668	0 0 2	27.70
3	35.38328	2.53475	1 1 -1	77.79
4	38.57241	2.33220	1 1 1	100.00
5	38.89273	2.31373	2 0 0	21.23
6	46.07489	1.96839	1 1 -2	2.09
7	48.63513	1.87059	2 0 -2	29.06
8	51.18811	1.78313	1 1 2	1.37
9	53.10608	1.72316	0 2 0	10.67
10	56.34233	1.63161	0 2 1	0.85
11	58.20054	1.58387	2 0 2	15.17
12	61.31937	1.51057	1 1 -3	20.44
13	65.42321	1.42539	0 2 2	16.40
14	66.08265	1.41276	3 1 -1	15.47

Tenorite

No.	2θ, °	d, Å	h k l	Norm. I.
15	66.33990	1.40790	3 1 0	0.46
16	67.69736	1.38293	1 1 3	9.29
17	67.74941	1.38200	2 2 0	14.40
18	68.52912	1.36816	2 2 -1	0.37
19	71.49936	1.31845	3 1 -2	0.33
20	72.24242	1.30670	3 1 1	7.36
21	72.59787	1.30118	2 2 1	0.29
22	74.79293	1.26834	0 0 4	5.93
23	74.85948	1.26738	2 2 -2	6.33
24	79.31650	1.20697	0 2 3	0.20
25	79.94840	1.19901	2 0 -4	2.01
26	79.98391	1.19857	1 1 -4	0.21
27	82.12568	1.17265	3 1 -3	4.77
28	82.68738	1.16610	2 2 2	4.55
29	83.31221	1.15894	3 1 2	0.18
30	83.49493	1.15686	4 0 0	4.06
31	86.32284	1.12607	2 2 -3	0.15
32	86.40105	1.12525	4 0 -2	1.59
33	87.40160	1.11493	1 3 0	0.00
34	87.70637	1.11184	1 1 4	0.16
35	89.08509	1.09817	1 3 -1	6.69
36	91.00322	1.07995	1 3 1	2.76
37	95.35194	1.04186	2 0 4	1.23
38	96.06241	1.03603	1 3 -2	0.00
39	97.78901	1.02229	3 1 -4	0.12
40	97.85818	1.02175	2 2 3	0.11
41	97.89490	1.02147	0 2 4	2.93
42	99.45562	1.00959	3 1 3	4.32
43	99.93692	1.00602	1 3 2	0.00

Table S7: Additional XRD data of the complex CuO-Au NPs contains characteristic peaks corresponding to the databases of each structure, monoclinic CuO and cubic Au crystal structures, and lattice parameters.

CuO-Ag_2O NP heterostructure

No.	2θ, °	d, Å	Height, cps	FWHM, °	Int. I., cps°	Int. W., °	Asymmetry	Decay(ηL/mL)	Decay(ηH/mH)	Size, Å
2	15.25(5)	5.80(2)	234(6)	0.62(11)	304(23)	1.30(13)	2.3(13)	1.5(2)	1.5(4)	135(24)
3	27.341(3)	3.2593(3)	11520(151)	0.154(2)	2476(27)	0.215(5)	0.94(7)	0.89(5)	0.58(5)	556(8)
4	27.95(9)	3.189(10)	219(5)	2.19(15)	672(41)	3.1(3)	0.94(7)	0.89(5)	0.58(5)	39(3)
5	31.767(2)	2.81453(18)	25160(242)	0.155(3)	6024(25)	0.239(3)	1.04(7)	0.88(3)	1.04(4)	555(10)
6	35.122(5)	2.5530(4)	8857(117)	0.583(5)	6744(39)	0.761(14)	1.72(7)	0.47(2)	0.74(4)	149.4(12)
7	37.682(9)	2.3852(5)	4133(64)	0.50(3)	2777(285)	0.67(8)	0.66(4)	0.70(3)	0.563(19)	174(11)
8	38.186(4)	2.3549(2)	9651(121)	0.613(10)	7869(296)	0.82(4)	0.66(4)	0.70(3)	0.563(19)	143(2)
9	43.811(10)	2.0647(5)	930(20)	0.40(2)	640(14)	0.69(3)	0.55(5)	1.55(13)	0.87(11)	222(11)
10	45.8041(12)	1.97940(5)	15338(182)	0.1785(17)	4041(12)	0.263(4)	1.21(4)	0.87(2)	0.86(2)	505(5)
11	47.037(17)	1.9304(7)	195(4)	0.25(6)	64(10)	0.33(6)	0.89(4)	0.88(5)	0.33(4)	367(83)
12	48.348(8)	1.8810(3)	2441(46)	0.769(8)	2499(21)	1.02(3)	0.89(4)	0.88(5)	0.33(4)	118.2(12)
13	53.116(9)	1.7229(3)	771(16)	0.683(16)	654(13)	0.85(3)	1.69(11)	0.00(9)	0.96(8)	136(3)
14	54.414(3)	1.68480(7)	4303(74)	0.184(3)	1095(10)	0.254(7)	1.62(11)	0.60(5)	0.88(6)	508(9)
15	57.076(3)	1.61238(7)	3832(65)	0.190(4)	969(23)	0.253(10)	1.60(10)	0.21(6)	1.08(6)	496(10)
16	57.64(2)	1.5980(5)	1133(23)	1.43(3)	2148(24)	1.90(6)	1.60(10)	0.21(6)	1.08(6)	66.4(12)
17	61.071(13)	1.5161(3)	1877(36)	0.808(13)	1968(23)	1.05(3)	0.96(7)	0.96(6)	0.00(7)	119.2(19)
18	64.138(11)	1.4508(2)	611(13)	0.39(3)	262(15)	0.43(3)	2.20(13)	0.00(6)	0.28(8)	251(19)
19	65.817(11)	1.4178(2)	2021(37)	1.007(14)	2232(33)	1.10(4)	2.20(13)	0.00(6)	0.28(8)	98.2(13)
20	67.575(14)	1.3851(3)	1835(34)	1.15(2)	2311(38)	1.26(4)	2.20(13)	0.00(6)	0.28(8)	87.0(15)
21	71.75(4)	1.3144(6)	529(11)	0.81(3)	458(21)	0.87(6)	0.92(17)	0.0(2)	0.0(2)	126(4)
22	74.088(6)	1.27865(9)	1040(21)	0.272(18)	359(26)	0.35(3)	0.91(7)	0.89(7)	0.00(12)	382(25)
23	74.699(17)	1.2697(2)	860(18)	0.92(4)	1005(31)	1.17(6)	0.91(7)	0.89(7)	0.00(12)	113(5)
24	76.356(3)	1.24622(4)	3533(63)	0.199(4)	894(31)	0.253(13)	0.91(7)	0.89(7)	0.00(12)	530(10)
25	76.85(2)	1.2394(3)	635(14)	0.71(6)	573(40)	0.90(8)	0.91(7)	0.89(7)	0.00(12)	149(12)

No.	2θ, °	Phase Name	Chemical Formula	Card No	Norm. I.	Profile Type	Distributi...
2	15.25(5)	Unknown			3.86	Split pseudo-Voigt	-
3	27.341(3)	Unknown			31.46	Split pseudo-Voigt	-
4	27.95(9)	Unknown			8.54	Split pseudo-Voigt	-
5	31.767(2)	Unknown			76.56	Split pseudo-Voigt	-
6	35.122(5)	Tenorite: 0 0 2	Cu O	1011194	85.71	Split pseudo-Voigt	-
7	37.682(9)	Silver oxide: 2 0 0	Ag2 O	1010486	35.29	Split pseudo-Voigt	-
8	38.186(4)	Tenorite: 1 1 1	Cu O	1011194	100.00	Split pseudo-Voigt	-
9	43.811(10)	Unknown			8.14	Split pseudo-Voigt	-
10	45.8041(12)	Tenorite: 1 1 -2	Cu O	1011194	51.35	Split pseudo-Voigt	-
11	47.037(17)	Unknown			0.81	Split pseudo-Voigt	-
12	48.348(8)	Tenorite: 2 0 -2	Cu O	1011194	31.76	Split pseudo-Voigt	-
13	53.116(9)	Tenorite: 0 2 0	Cu O	1011194	8.31	Split pseudo-Voigt	-
14	54.414(3)	Silver oxide: 2 2 0	Ag2 O	1010486	13.91	Split pseudo-Voigt	-
15	57.076(3)	Unknown			12.32	Split pseudo-Voigt	-
16	57.64(2)	Tenorite: 2 0 2,Silver oxide: 2...	Cu O,Ag2 O	1011194,1010486	27.29	Split pseudo-Voigt	-
17	61.071(13)	Tenorite: 1 1 -3,Silver oxide:...	Cu O,Ag2 O	1011194,1010486	25.01	Split pseudo-Voigt	-
18	64.138(11)	Unknown			3.33	Split pseudo-Voigt	-
19	65.817(11)	Tenorite: 3 1 0	Cu O	1011194	28.37	Split pseudo-Voigt	-
20	67.575(14)	Tenorite: 2 2 0,Silver oxide: 2...	Cu O,Ag2 O	1011194,1010486	29.37	Split pseudo-Voigt	-
21	71.75(4)	Tenorite: 2 2 1	Cu O	1011194	5.82	Split pseudo-Voigt	-
22	74.088(6)	Tenorite: 0 0 4	Cu O	1011194	4.57	Split pseudo-Voigt	-
23	74.699(17)	Silver oxide: 3 2 1	Ag2 O	1010486	12.77	Split pseudo-Voigt	-
24	76.356(3)	Unknown			11.36	Split pseudo-Voigt	-
25	76.85(2)	Unknown			7.28	Split pseudo-Voigt	-

Lattice parameters

Phase name	a, Å	b, Å	c, Å	α, °	β, °	γ, °
Tenorite	4.73589	3.47839	5.19224	90.000	99.530	90.000
Silver oxide	4.77575	4.77575	4.77575	90.000	90.000	90.000

d-I List

Tenorite

No.	2θ, °	d, Å	h k l	Norm. I.
1	32.05744	2.78972	1 1 0	7.38
2	35.01888	2.56029	0 0 2	27.61
3	35.04634	2.55835	1 1 -1	77.60
4	38.20282	2.35391	1 1 1	100.00
5	38.51983	2.33527	2 0 0	21.18
6	45.62586	1.98672	1 1 -2	2.11
7	48.15802	1.88800	2 0 -2	29.20
8	50.68244	1.79972	1 1 2	1.38
9	52.57856	1.73920	0 2 0	10.71
10	55.77711	1.64680	0 2 1	0.86
11	57.61316	1.59861	2 0 2	15.29
12	60.69390	1.52463	1 1 -3	20.62
13	64.74574	1.43866	0 2 2	16.56
14	65.39661	1.42591	3 1 -1	15.61
15	65.65049	1.42101	3 1 0	0.46
16	66.99005	1.39581	1 1 3	9.37
17	67.04141	1.39486	2 2 0	14.53
18	67.81070	1.38090	2 2 -1	0.37
19	70.74039	1.33072	3 1 -2	0.34
20	71.47307	1.31887	3 1 1	7.43
21	71.82351	1.31329	2 2 1	0.29
22	73.98721	1.28015	0 0 4	5.99
23	74.05279	1.27917	2 2 -2	6.39

Tenorite

No.	2θ, °	d, Å	h k l	Norm. I.
24	78.44317	1.21820	0 2 3	0.21
25	79.06529	1.21017	2 0 -4	2.03
26	79.10025	1.20973	1 1 -4	0.22

Silver oxide

No.	2θ, °	d, Å	h k l	Norm. I.
1	26.37086	3.37696	1 1 0	1.63
2	32.44502	2.75728	1 1 1	100.00
3	37.63887	2.38787	2 0 0	42.29
4	46.54274	1.94969	2 1 1	0.47
5	54.28520	1.68848	2 2 0	35.24
6	57.87827	1.59192	2 2 1	0.00
7	61.33492	1.51022	3 1 0	0.15
8	64.68100	1.43994	3 1 1	34.67
9	67.93696	1.37864	2 2 2	8.73
10	74.24263	1.27637	3 2 1	0.13

Table S8: Additional XRD data of the complex CuO-Ag_2O NPs contains characteristic peaks corresponding to the databases of each structure, monoclinic CuO and cubic Ag_2O crystal structures, and lattice parameters.

3.6. Electron microscopy (EM) - additional data

3.6.1. Monometalic NPs - control

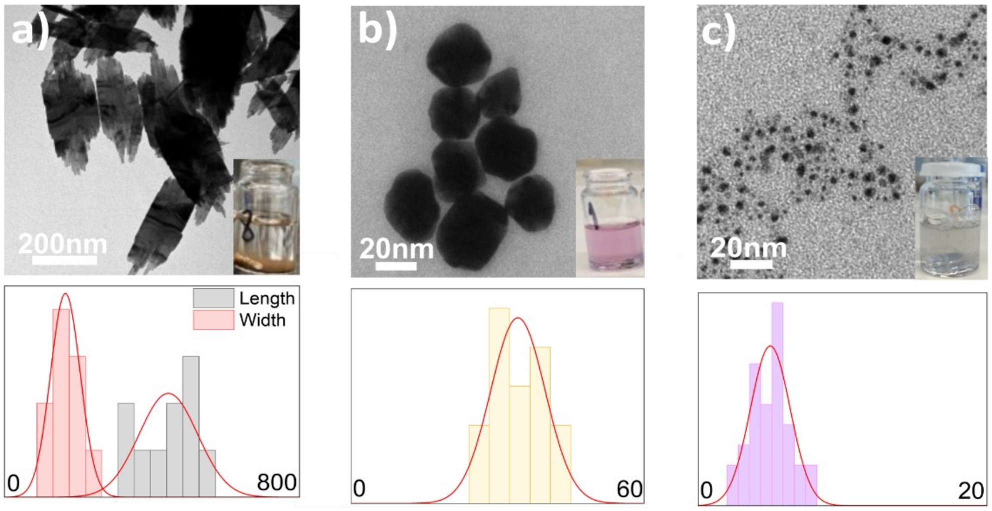

Figure S12: SEM images and size distributions of monometallic nanoparticles (controls). (a) Copper acetate produced a brown solid corresponding to CuO, with an average length of 439±70nm and a width of 171±39 nm. **(b)** Chloroauric acid yielded a pink suspension of spherical AuNPs with an average diameter of 34±5nm. **(c)** Silver nitrate generated a thin black precipitate after several days at room temperature, forming dot-like Ag2O NPs with an average size of ~5nm.

3.6.2. CuO-Au NP heterostructure

Additional SEM images and atomic distribution data for the samples are presented in the figure below. The analysis revealed monodispersed nanoparticles composed of three elements: Au, Cu, and O, confirming the formation of the CuO-Au NP heterostructure.

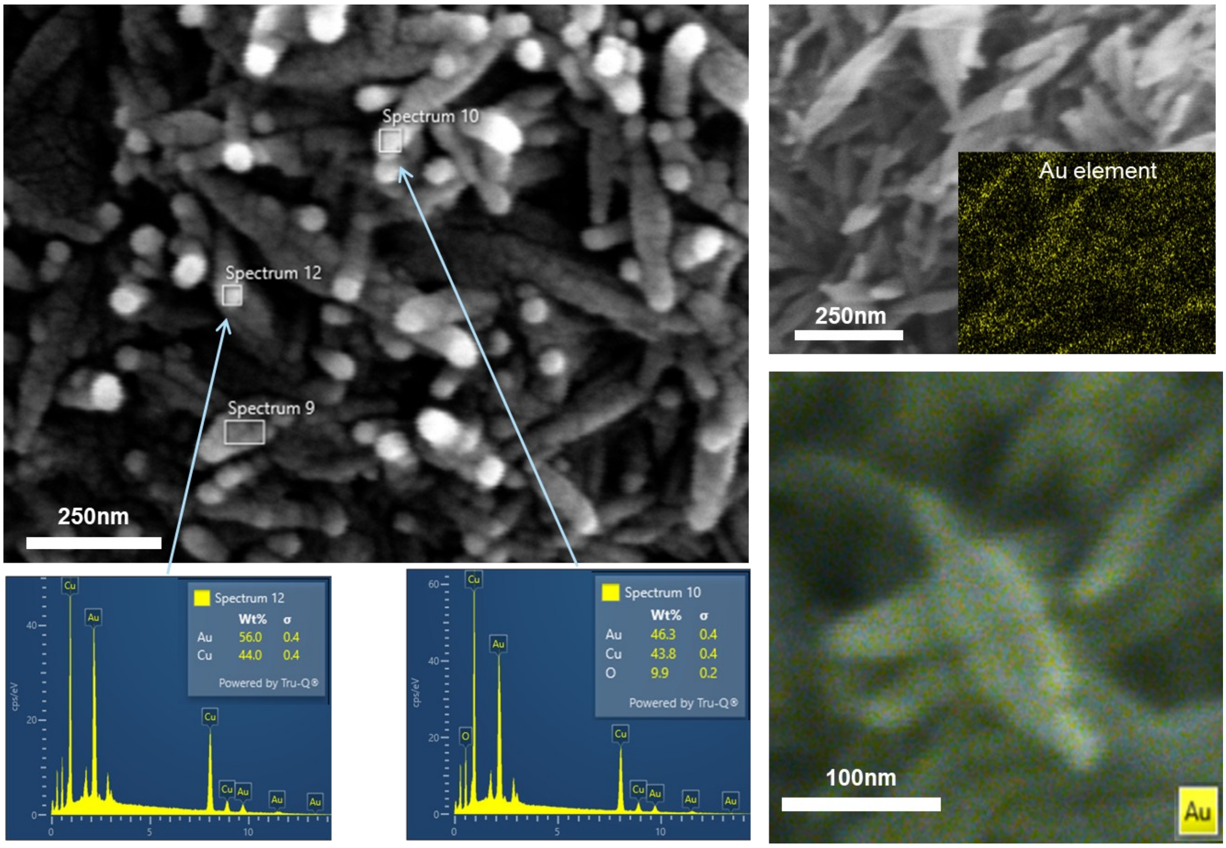

Figure S13: SEM images reveal two distinct nanostructures: a leaf-like morphology corresponding to the CuO core material, and spherical nanoparticles decorating its surface. EDX analysis confirms that the leaf-like nanostructure contains copper (Cu) and oxygen (O), while the spherical NPs are composed of gold (Au). EDX mapping further shows the distribution of Au along the CuO nano-leaves, with aggregation appearing as dominant yellow spots at the particle edges.

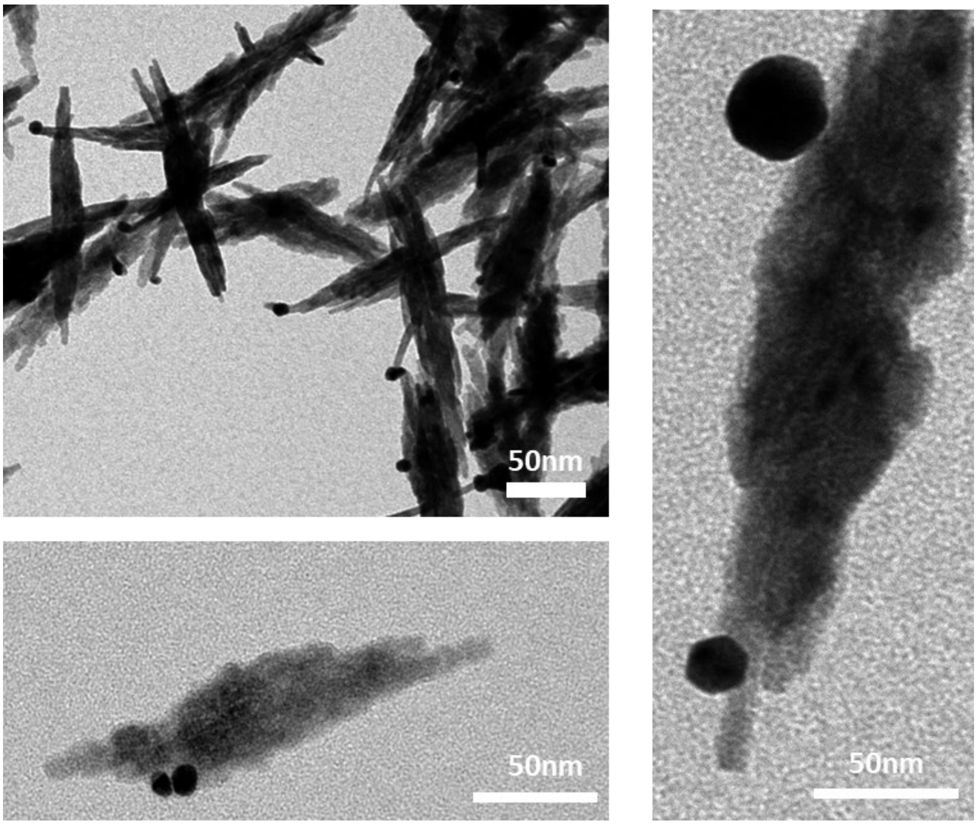

Figure S14: TEM images provide a detailed visualization of the distribution of gold nanoparticles (AuNPs) on the CuO nano-leaf surface. The AuNPs are located mostly along the CuO nano-leaf tips.

3.6.3. CuO-Ag_2O NP heterostructure

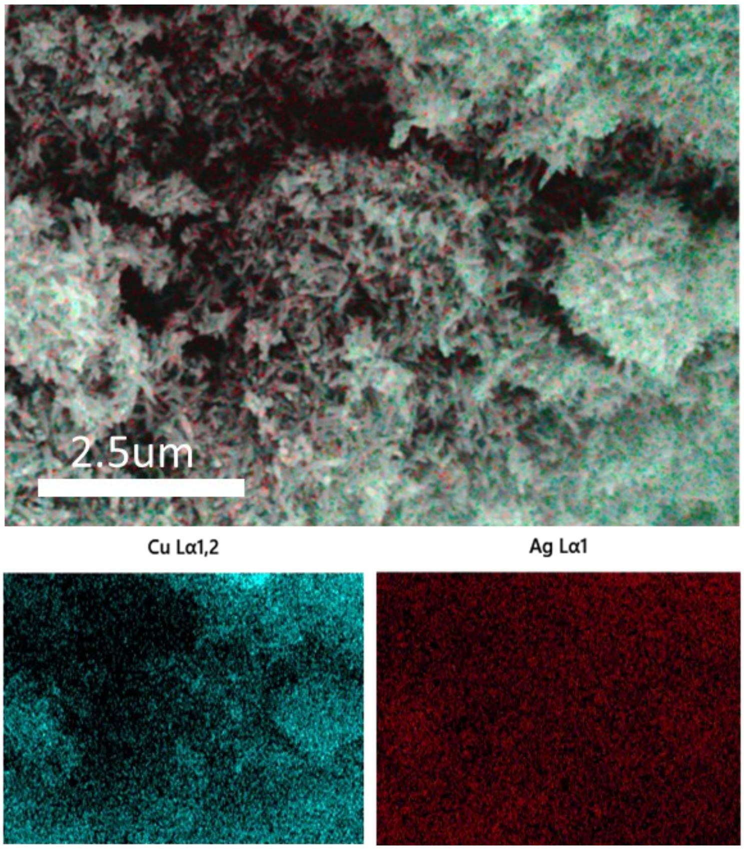

Figure S15: SEM image and EDX elemental mapping analysis confirm that the leaf-like nanostructure consists of copper (blue) and oxygen (green), characteristic of the core CuO. The surface is decorated with small spherical nanoparticles composed of silver (red), indicating successful formation of the CuO–Ag_2O heterostructure.

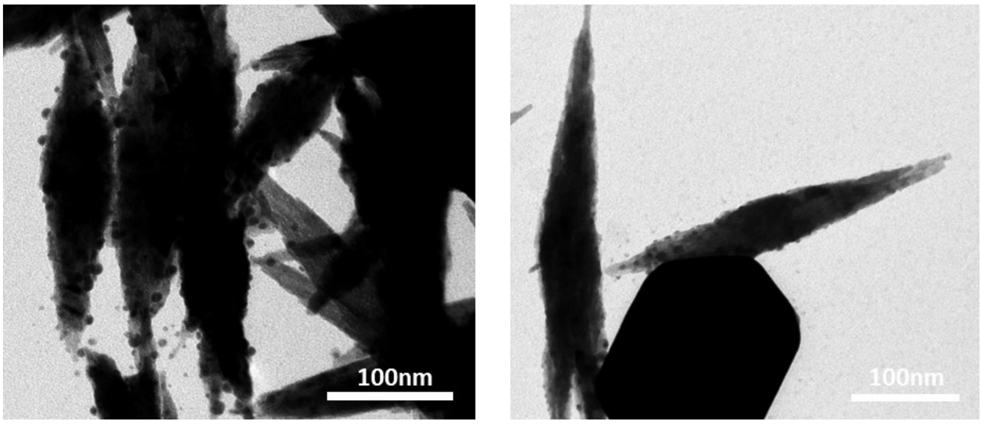

Figure S16: TEM images provide a detailed visualization of the distribution of silver oxide (Ag_2O) NPs on the CuO nano-leaf surface. The Ag_2O NPs are distributed along the CuO surface.

3.7. Inductively Coupled Plasma Optical Emission Spectroscopy (ICP-OES)

8.75 mg of NP was suspended in 0.25 mL type 1 DI water, 2 mL of ICP grade HNO_3 was added, and the solution was warmed to a boil. On digestion and after cooling, a further 5 mL of type 1 DI water was added via pipette to wash down the sides of the beaker and watch glass. The resulting solution was transferred quantitatively with type 1 DI water to a 50 mL Class A Volumetric flask and made up to the mark after inversion. The resulting solution was transferred to a clean 50 mL centrifuge tube for analysis. Additionally, five calibration standards were prepared from a multi-element calibration standard solution: 1, 5, 10, 25, and 50 ppm for further analysis.

Sample	Element	Avg. ppm	Mass (mg)	wt%	mol (in 50mL)	Molar Ratio (Cu: Metal)
**CuO-Au**	Cu	7.29	0.3645	4.17	5.74×10-6	
	Au	0.107	0.00535	0.0611	2.72×10-8	**211 : 1**
**CuO-Ag_2O**	Cu	12.63	0.6315	7.22	9.94×10-6	
	Ag	0.05	0.0025	0.0286	2.32×10-8	**428 : 1**

Table S9: Elemental analysis of nanoparticle (NP) heterostructures measured by ICP-OES, including the weight percent (wt%) of each element and the molar ratio of Cu to the noble metal (Au or Ag).

3.8. Photodegradation test- Control samples

To further clarify the role of noble-metal coupling in the CuO-Au and CuO-Ag_2O hybrids, we performed additional control experiments using unsupported Au NPs and Ag_2O. These tests were designed to evaluate whether the observed photocatalytic enhancement could arise solely from noble-metal activity, independent of the CuO host. To this end, we conducted testing of <1mg quantities (approximating the noble-metal loading present in the CuO hybrids). The UV-Vis spectra are presented below:

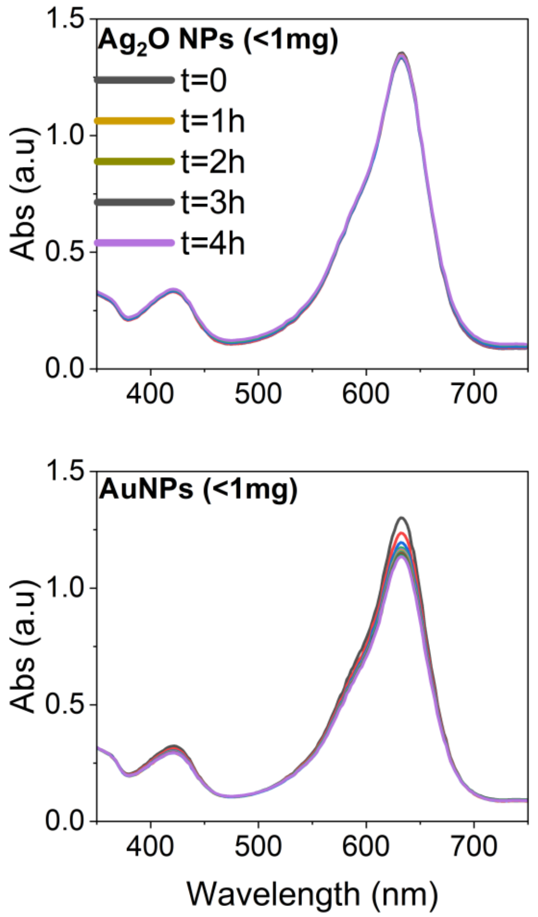

Figure S17: UV-Vis absorbance spectra of unsupported noble-metal nanoparticles during photodegradation of MG. Ag_2O NPs and Au NPs showed minimal dye degradation between 5% to 12%.

4. References

(1) Phetsahai, A.; Eiamchai, P.; Thamaphat, K.; Limsuwan, P. The Morphological Evolution of Self-Assembled Silver Nanoparticles under Photoirradiation and Their SERS Performance. *Processes* **2023**, *11* (7). https://doi.org/10.3390/pr11072207.

(2) Métraux, G. S.; Cao, Y. C.; Jin, R.; Mirkin, C. A. Triangular Nanoframes Made of Gold and Silver. *Nano Lett* **2003**, *3* (4). https://doi.org/10.1021/nl034097+.
